# Single-cell transcriptomic analysis reveals a novel cell state and switching genes during hepatic stellate cell activation in vitro

**DOI:** 10.1186/s12967-022-03263-4

**Published:** 2022-01-29

**Authors:** Hua Wang, Shaoping Zheng, Hongbo Jiang, Xuejia Wang, Fengqin Zhou, Zhihong Weng

**Affiliations:** 1 Department of Infectious Diseases, Union Hospital, Tongji Medical College, Huazhong University of Science and Technology, 1277 JieFang Avenue, Wuhan, 430022 China; 2 Department of Ultrasound, Union Hospital, Tongji Medical College, Huazhong University of Science and Technology, Wuhan, China; 3 Department of Epidemiology and Biostatistics, School of Public Health, Guangdong Pharmaceutical University, Guangzhou, China

**Keywords:** Liver fibrosis, Hepatic stellate cell, Single-cell RNA sequencing

## Abstract

**Background:**

The transformation of hepatic stellate cell (HSC) to myofibroblast is a key event during liver fibrogenesis. However, the differentiation trajectory of HSC-to-myofibroblast transition and the switching genes during this process remains not well understood.

**Methods:**

We applied single-cell sequencing data to reconstruct a single-lineage pseudotime trajectory of HSC transdifferentiation in vitro and analyzed the gene expression patterns along the trajectory. GeneSwitches was used to identify the order of critical gene expression and functional events during HSC activation.

**Results:**

A novel cell state during HSC activation was revealed and the HSCs belonging to this state may be an important origin of cancer-associated fibroblasts (CAFs). Combining single-cell transcriptomics with GeneSwitches analyses, we identified some distinct switching genes and the order at which these switches take place for the new state of HSC and the classic culture-activated HSC, respectively. Based on the top switching genes, we established a four-gene combination which exhibited highly diagnostic accuracy in predicting advanced liver fibrosis in patients with nonalcoholic fatty liver disease (NAFLD) or hepatitis B (HBV).

**Conclusion:**

Our study revealed a novel cell state during HSC activation which may be relevant to CAFs, and identified switching genes that may play key roles in HSC transdifferentiation and serve as predictive markers of advanced fibrosis in patients with chronic liver diseases.

**Supplementary Information:**

The online version contains supplementary material available at 10.1186/s12967-022-03263-4.

## Introduction

Hepatic fibrosis is a common wound-healing response to diverse chronic liver diseases. The pathogenesis of hepatic fibrosis is mostly featured by excessive accumulation of extracellular matrix (ECM) in the liver [1], which is mainly derived from activated hepatic stellate cell (HSC). Upon different kinds of hepatic injuries, quiescent HSCs are activated and transformed into proliferative, fibrogenic and contractile myofibroblasts, which are characterized by expressing high level of *Acta2* (smooth muscle actin alpha 2). HSC activation is recognized as a pivotal event during liver fibrogenesis [2, 3].

Single-cell transcriptomic analysis is widely used in basic scientific and clinical researches, and has reshaped our understanding of many complex biological processes. Compared with traditional sequencing methods, single-cell RNA sequencing (scRNA-seq) can offer unprecedented insight into HSC heterogeneity and identify genes highly related to HSC activation at single-cell resolution. Recently, HSC zonation has been proposed as a determinant of the liver fibrogenesis response [4]. The subsets of HSC and myofibroblast during hepatic fibrosis have been identified by single-cell transcriptomic analysis [5]. However, the differentiation trajectory of HSC-to-myofibroblast transition and the switching genes during this process remains not well understood.

In the present study, we in silico reconstructed a single-lineage pseudotime trajectory of HSC activation in vitro based on the scRNA-seq data. Intriguingly, we identified a novel cell state during HSC transdifferentiation, which may be relevant to cancer-associated fibroblasts (CAFs). In addition, we dissected key switching genes contributing to HSC activation, then, we established a four-gene combination based on the top switching genes and evaluated its diagnostic accuracy in predicting advanced liver fibrosis in humans.

## Materials and methods

### scRNA-seq data analysis

Published scRNA-seq data were retrieved from the Gene Expression Omnibus (GEO) dataset GSE132662 [5]. It includes single-cell transcriptomic data of the primary HSCs which were isolated from healthy mouse liver, cultured in vitro and harvested at day 0, 1, 3, 7, and 9. The scRNA-seq downstream analyses were performed using the R package Seurat 3.0 [6]. Low quality cells (< 200 genes/cell, > 6000 genes/cell, < 3 cells/gene and > 20% mitochondrion genes) were filtered out from the dataset. Then, the gene expression in the remained cells was normalized using a linear regression model. Highly variable genes were identified and selected for principal component analysis (PCA) to identify significantly available dimensions [7]. Afterwards, the t-distributed stochastic neighbor embedding (t-SNE) algorithm was applied for performing dimensionality reduction and cluster analysis [8].

To dissect the activation process of quiescent HSC in vitro at single-cell resolution, we used Monocle2 [9] to perform in silico pseudotime trajectory analysis of HSC transdifferentiation. Briefly, the “DDRTree” reduction method was used and single cells were ordered into a trajectory with branch points. The cells in different branches were considered to be in the different cell differentiation state. Genes showed differential expression levels between branches were defined as state-specific genes.

### GeneSwitches analysis

To discover the order of critical gene expression and functional events during HSC activation, we apply GeneSwitches [10], a tool that can process scRNA-seq data together with pseudotime trajectories to identify the genes (named switching genes) that act as on/off switches between cell states and importantly the ordering at which these switches take place. GeneSwitches first binarizes the input gene into either an “on” or “off” gene-expression state to facilitate the identification of switching events. For each gene in each cell, the binarized gene expression is used as a dependent variable in logistic regression with the pseudotime value of each cell providing the independent variable. Using this, GeneSwitches calculates the probability of gene-expression throughout pseudotime and estimated the quality of fit using McFadden’s Pseudo *R*^2^ [11]. The activated switching genes positively correlated with pseudotime (*R*^2^ > 0) were defined as upregulation genes, while the silenced switching genes negatively correlated with pseudotime (*R*^2^ < 0) were defined as downregulation genes. The higher the pseudotemporal correlation, the closer the relationship between switching genes and the trajectory process.

In addition, GeneSwitches includes the pathways provided by Molecular Signatures Database (MSigDB) hallmark [12], C2 Kyoto Encyclopedia of Genes and Genomes (KEGG) [13] and C5 gene ontology gene set collections and can be used to order pathways or gene sets (e.g., functional ontologies). GeneSwitches can also compare the ordering of switching genes from two related pseudotime trajectories. The common switching genes between two trajectories can be plotted. Moreover, GeneSwitches can identify and plot the distinct switching genes that are specific to each trajectory [10].

### Human and mouse expression array

Expression data from two studies of patients with nonalcoholic fatty liver disease (NAFLD) and a study of patients with hepatitis B (HBV) staged for liver fibrosis, respectively, were retrieved from the GEO database GSE49541 [14], GSE89632 [15], and GSE84044 [16].

Bulk RNA sequencing data were obtained from dataset GSE154170 [17], which performed in 4 types of mouse samples: isolates of quiescent HSC, isolates of HSC from bile duct-ligated (BDL) mice biliary fibrosis model, HSC isolates from mice treated with 3,5-diethoxycarbonyl-1,4-dihydrocollidine (DDC) diet biliary fibrosis model, and isolates of HSC-derived CAF from YAP/AKT or KRAS/sg-p19 induced intrahepatic cholangiocarcinoma (CCA).

### Cell culture and immunofluorescence

Human hepatic stellate LX2 cells were cultured in Dulbecco’s modified Eagle’s medium (DMEM) (Invitrogen, USA) supplemented with 10% fetal bovine serum (Gibco, USA). Recombinant human TGF-β1 was acquired from R&D Systems (USA). The antibody against *TAGLN* was purchased from Cell Signaling Technology (USA). Integrin αv (*ITGAV*) antibody was purchased from BD Biosciences (USA). The fluorescent secondary antibodies were conjugated with Alexa Fluor 594 (red) or Alexa Fluor 488 (green) (Invitrogen, USA). All samples were examined using a fluorescence microscope (Nikon, Japan).

### Statistical analysis

Kruskal–Wallis test in R (version 4.1.0) was applied for comparisons among groups. The ClueGO (version 2.5.8) and CluePedia (version 1.5.8) plugins in Cytoscape (https://cytoscape.org/), were used to perform functional enrichment analysis of the identified genes. The area under the receiver operating characteristic curve (AUROC) was conducted using pROC [18]. Comparison of the paired samples from human were performed in R and visualized using ggplot2 [19]. P-values < 0.05 were considered as statistical significance. The development and validation of the prediction model were detailed in the Additional file 1.

## Result

### Single-cell analysis of HSCs isolated from healthy mice

**Fig. 1:**
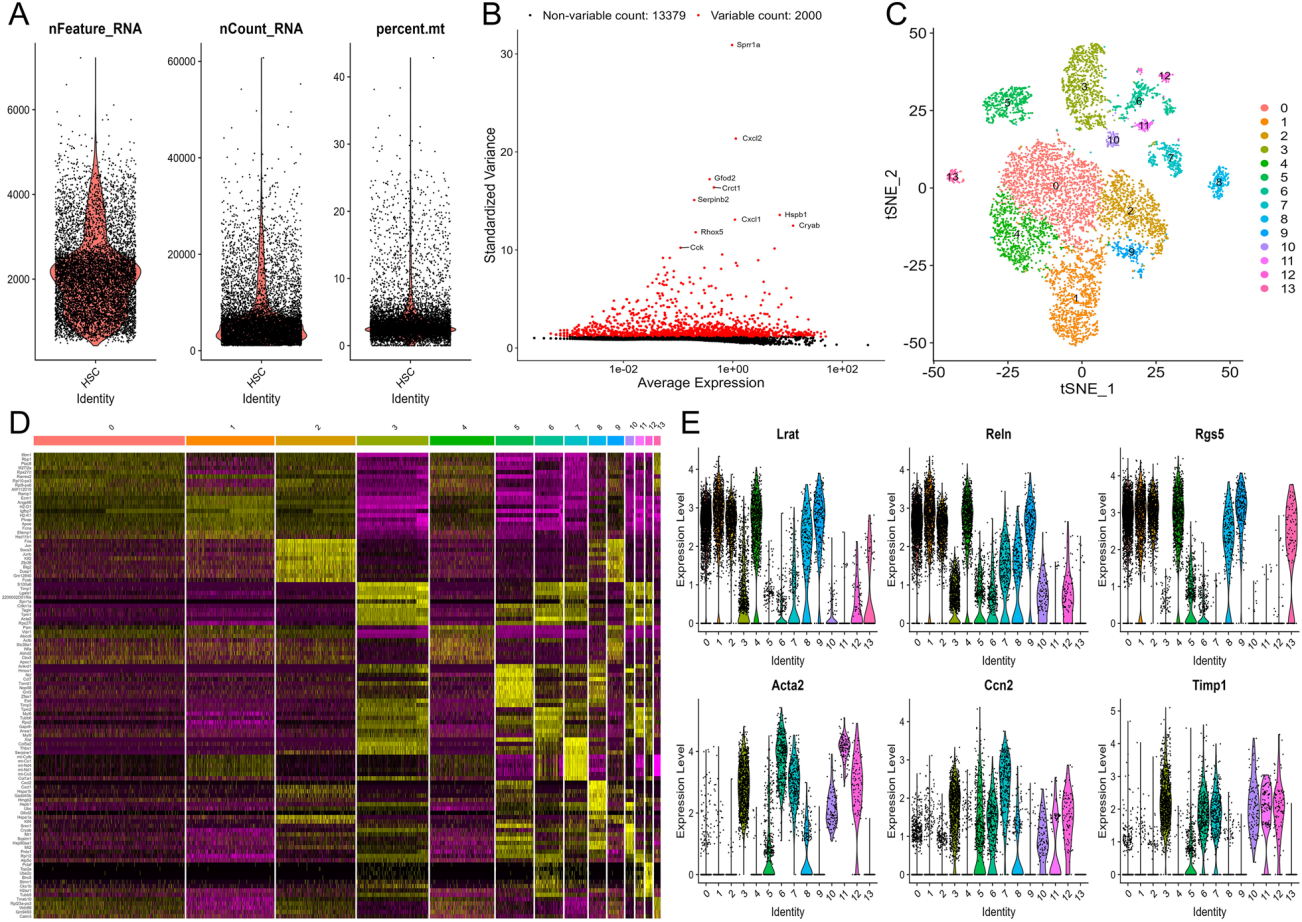
Identification of 14 cell clusters in the HSCs cultured in vitro based on single-cell RNA-seq data. **A** After quality control of the cells from mouse HSC samples cultured in vitro, 8500 cells were included in this analysis. **B** The top 10 highly variable genes are marked in the plot. **C** t-SNE plot displays 14 cell clusters in the HSCs. **D** The top 10 marker genes of each cell cluster are listed beside of a heatmap. The gene expression levels from low to high are showed by colors from purple to yellow. **E** Violin plot displays the expression of the marker genes of HSC

After the quality control and the normalization of scRNA-seq data (GSE132662) [5], 8500 cells and 15,379 genes were included in our analysis (Fig. 1A), and 2000 highly variable genes were identified for subsequent analysis (Fig. 1B). Then, the HSCs harvested at day 0, 1, 3, 7, and 9 in vitro were successfully classified into 14 separate clusters using t-SNE algorithm (Fig. 1C), and top marker genes from all 14 clusters were showed in Fig. 1D. Based on the expression patterns of the marker genes, cells in cluster 0, 1, 2, 4, 8, 9, and 13 were annotated as quiescent HSC, expressing high levels of quiescence markers such as *Lrat*, *Reln*, and *Rgs5* [20], while cells in cluster 3, 5, 6, 7, 10, 11, and 12 were annotated as activated HSC, expressing high activation markers *Acta2*, *Ccn2*, and *Timp1* (Fig. 1E).

### The differentiation trajectory analysis of HSCs

**Fig. 2:**
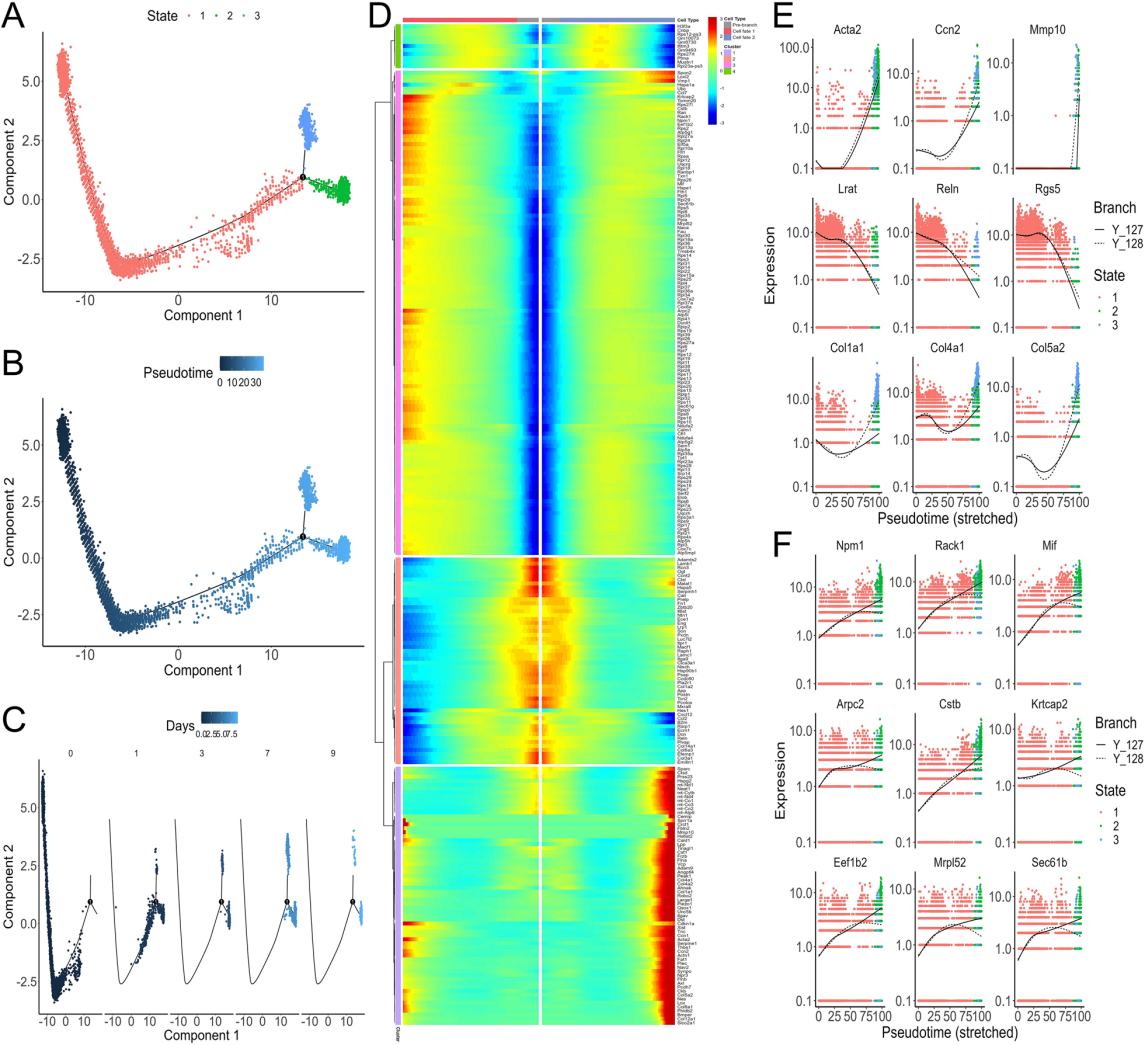
Simulation of the differentiation trajectory of HSC and the analysis of gene expression pattern. **A** Trajectory reconstruction of all single cells reveals two branches. HSCs are colored by states, which denote branches and can be used to extract paths. Path 1 consists of the cells in states 1 and 2, while path 2 contains the cells in the states 1 and 3. **B** HSCs are colored by pseudotime, showing cells developing from state 1 to the bifurcation point that gives rise to the final state 2 and 3, respectively. **C** HSCs harvested at day 0, 1, 3, 7, and 9 are plotted along pseudotemporal trajectories. **D** The branched heatmap shows the dynamics of top 250 differentially expressed genes between the two cell fate branches during HSC transdifferentiation. Genes (rows) are divided into four clusters and cells (columns) are ordered according to the pseudotime trajectory. **E**, **F** Expression patterns of selected genes between two cell fate branches. The full line represents branch 1 (state 1 and 2) while dotted line represents branch 2 (state 1 and 3)

Furthermore, we visualized the transcriptional profile of the HSCs and mapped them along pseudotemporal trajectories. Our data suggested a differentiation trajectory from quiescent HSC to activated HSC, enabling the allocation of 3 pseudotime-dependent differentiation states for HSC (Fig. 2A). Moreover, the pseudotime of HSC transdifferentiation was just in accordance with HSC culture-activation process in vitro (Fig. 2B, C). Notably, the HSCs bifurcated into two diverse branches (state 2 and 3) (Fig. 2A), representing two cell fates (cell fate 1 and 2) (Fig. 2D), respectively. From the perspective of cell sourcing, state 1 (pre-branch) was populated mainly by the HSCs harvested at day 0 and partly at day 1. Similar to that of state 2 (cell fate 1), the cell composition of state 3 (cell fate 2) were also from the HSCs harvested at day 3, 7, 9 and partly at day 1 (Fig. 2A, C, D). Therefore, state 2 and 3 may correspond to the status of HSC-to-myofibroblast transition. In order to gain insights into the process of HSC transdifferentiation, we performed a branched heatmap to show the gene expression patterns of these two cell fate branches based on the dynamics of top 250 differentially expressed genes. These genes were then divided into four clusters (Fig. 2D) according to their characterized patterns and listed in Additional file 2: Table S1.

**Fig. 3:**
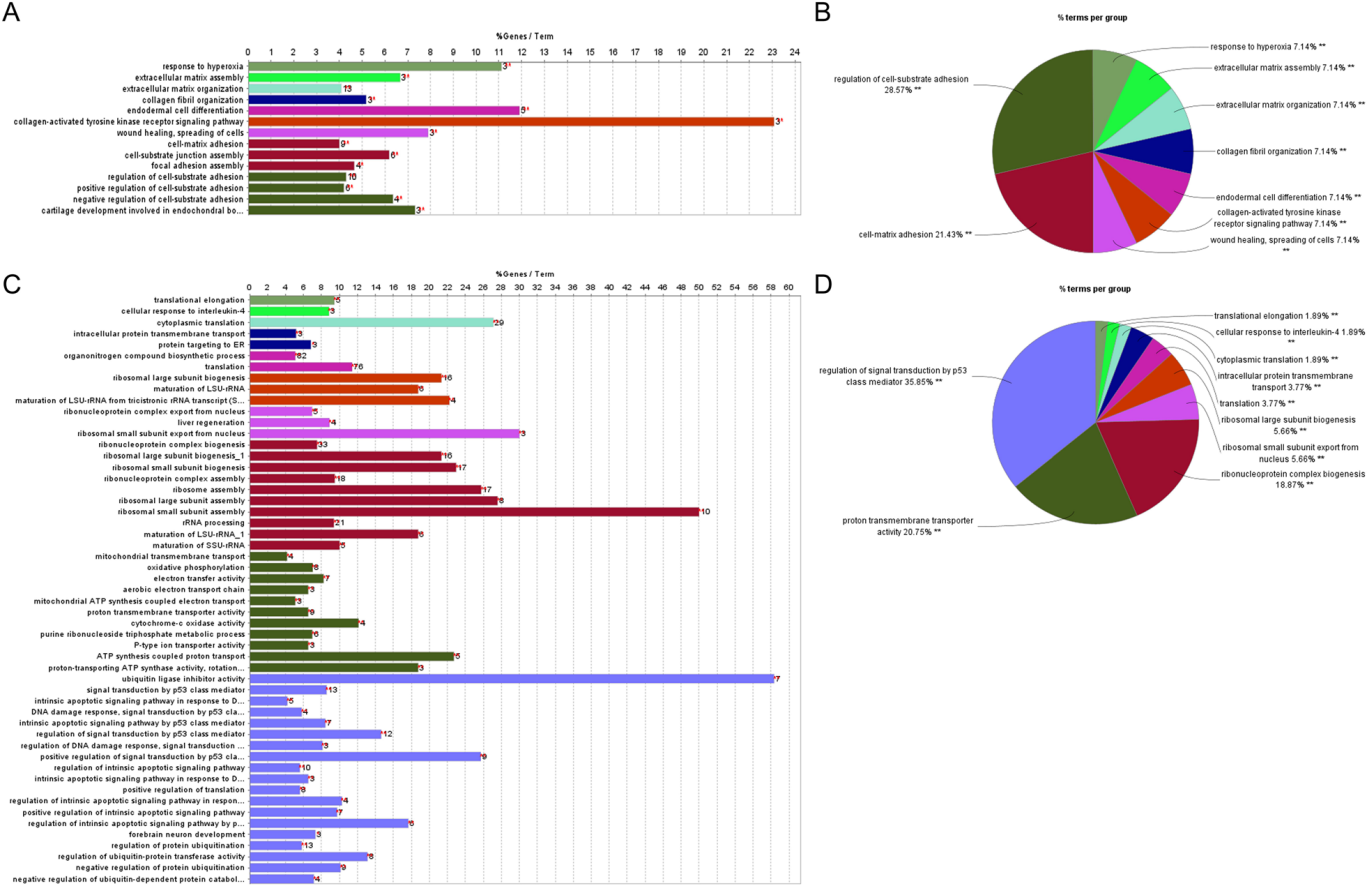
ClueGO and CluePedia were used for biological process annotation of the genes in cluster 1 and 3 of the branched heatmap for HSC differentiation trajectory. The bar chart shows GO terms specific for the genes in cluster 1 (**A**) and 3 (**C**). The number of genes relevant to the terms has been shown. The pie chart with functional groups shows main biological processes related to the genes in cluster 1 (**B**) and 3 (**D**). The different groups are assigned with individual colors. The groups are ordered according to the proportion of corresponding genes belonging to the groups. Two stars indicates to P-value < 0.001

As shown in Fig. 2D, the top member genes in cluster 1 were gradually upregulated from the beginning of pre-branch (state 1) and reached a high level at the final stage in cell fate 2 (state 3). Functional enrichment analysis was performed using ClueGO and CluePedia, which indicated that these genes largely enriched in the biological processes such as “regulation of cell-substrate adhesion”, “cell–matrix adhesion”, and “extracellular matrix assembly” (Fig. 3A, B). Concurrent with the high expression of activation markers of HSC (e.g., *Acta2*, *Ccn2*, *Mmp10*, *Col1a1*, *Col4a1*, and *Col5a2*), the expression levels of quiescence markers (*Lrat*, *Reln*, and *Rgs5*) in branch 2 (including state 1 and 3) rapidly fell off from the beginning of state 1 (Fig. 2E). Therefore, the cells in state 3 represent classic culture-activated HSCs (myofibroblasts) in vitro. Meanwhile, together with downregulated quiescence markers in branch 1 (state 1 and 2), the expression levels of *Acta2*, *Ccn2*, and *Mmp10*, markers of activated HSC, were also high in state 2 (Fig. 2E), suggesting that the HSCs belonging to state 2 may also be activated. However, some collagen genes in state 2 were expressed relative lower than those in state 3, such as *Col1a1*, *Col4a1*, *Col5a2* (Fig. 2E).

Interestingly, most ribosomal protein genes in cluster 3 were expressed higher in cell fate 1 (state 2) than those in cell fate 2 (state 3) (Fig. 2D), which indicates that the cells in state 2 may have higher translational capacity to accommodate HSC transdifferentiation and proliferation than those of the cells in state 3. Apart from ribosomal subunit genes, other genes in cluster 3 were gradually upregulated in state 1 while their expression was no longer increased, even downregulated in state 3, moreover, these genes were continuously upregulated along branch 1 (state 1 and 2) (e.g., *Npm1*, *Rack1*, *Mif*, *Cstb*, *Arpc2*, and *Krtcap2*, etc.) (Fig. 2F). Then, the genes in cluster 3 were selected for functional enrichment analysis, which revealed that the functional group with the highest percentage of corresponding genes was “regulation of signal transduction by p53 class mediator”, including “regulation of DNA damage response, signal transduction by p53 class mediator”, “intrinsic apoptotic signaling pathway by p53 class mediator”, and “regulation of protein ubiquitination” (Fig. 3C, D; Additional file 3: Table S2), all of which were closely related to tumors. Especially, some genes such as *Npm1*, *Rack1*, *Mif*, *Cstb*, and *Arpc2* were involved in liver carcinogenesis [21–25]. According to the differentiation trajectory analysis of HSC, we proposed that state 2 may be a specific state of HSC, which might be relevant to the regulation of tumorigenesis.

**Fig. 4:**
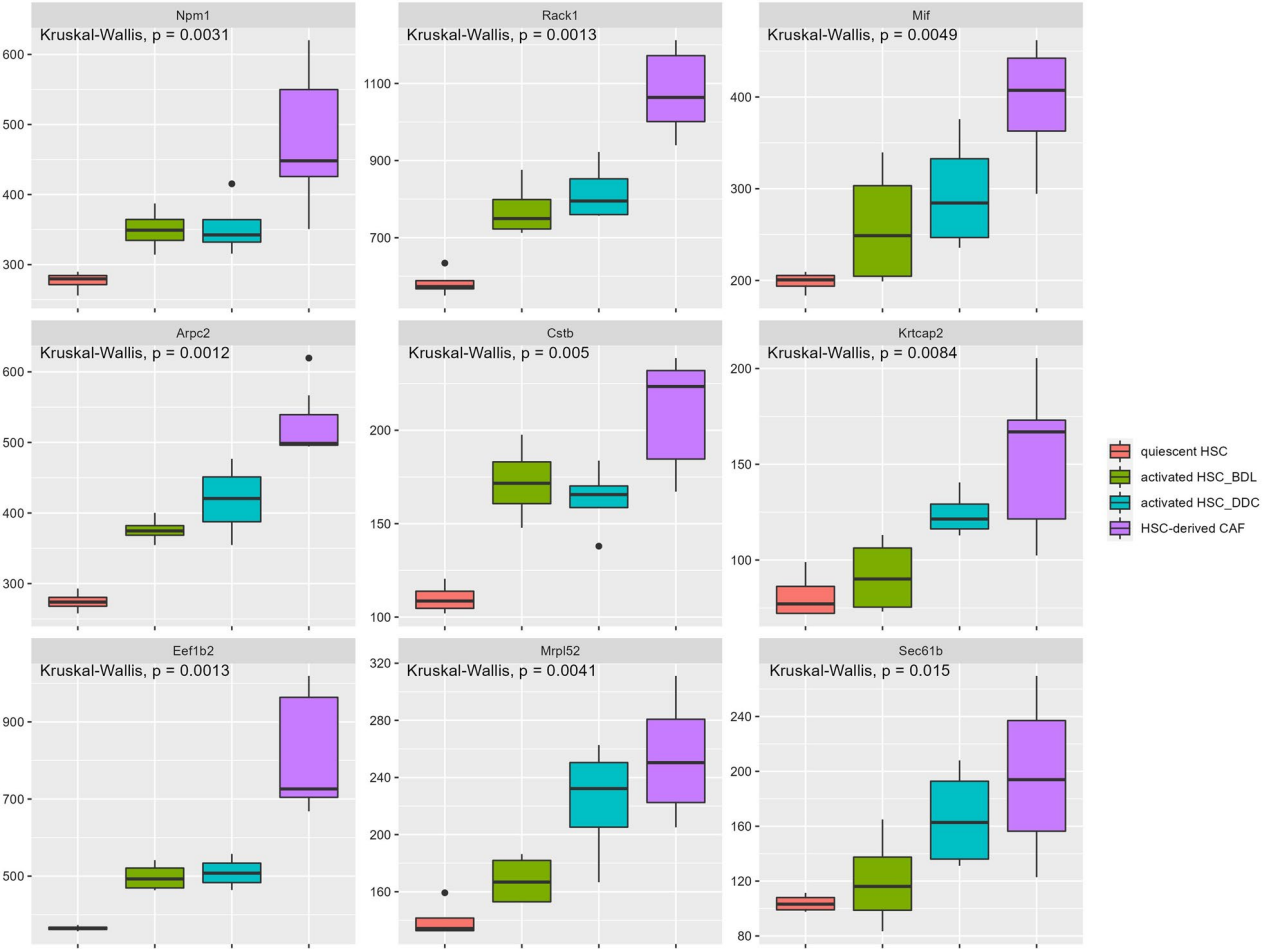
Visualization of the gene expression pattern of HSCs based on bulk RNA-sequencing data from dataset GSE154170. Kruskal-Wallis test examined differences in gene expression among 4 groups of mouse samples which includes: isolates of quiescent HSC, isolates of HSC from bile duct-ligated (BDL) mice biliary fibrosis model, HSC isolates from mice treated with 3,5-diethoxycarbonyl-1,4-dihydrocollidine (DDC) diet biliary fibrosis model, and isolates of HSC-derived CAF from intrahepatic cholangiocarcinoma (CCA) model. P < 0.05 was considered as statistically significant

It has been demonstrated that CAFs derived from activated HSCs constitute the major population in the hepatocellular carcinoma (HCC) stroma and positively influence HCC progression [26]. We then analyzed bulk RNA-sequencing data from dataset GSE154170 including gene expression profiles of quiescent HSCs, activated HSCs from BDL or DDC diet biliary fibrosis model, and HSC-derived CAFs from CCA model. The results showed that genes such as *Npm1*, *Rack1*, *Mif*, *Cstb*, *Arpc2*, and *Krtcap2* were expressed much higher in HSC-derived CAFs than those in activated HSCs from BDL or DDC model (Fig. 4). These cancer-related genes expressed in the HSCs belonging to state 2 seemed to share similar expression features with those in the HSC-derived CAFs (Fig. 2F). Therefore, we inferred that the HSCs in state 2 may be an important origin of CAFs in liver tumor microenvironment (TME).

Additionally, to gain insight into the activation of HSCs in vivo, we analyzed a scRNA-seq dataset GSE137720 [4] of HSCs isolated from healthy and CCl_4_-treated mouse liver. As shown in Additional file 4: Fig. S1, the differentiation trajectory of the HSCs also revealed two branches. The cell composition of state 1 were mainly from quiescent HSCs, state 2 (cell fate 1) corresponded to the status of HSC-to-myofibroblast transition, and state 3 (cell fate 2) may be the novel state of HSC. Functional enrichment analysis indicated that the top genes expressed in the HSCs belonging to state 3 enriched in the biological processes such as “ubiquitin ligase inhibitor activity”, “ribosomal large subunit biogenesis”, “signal transduction by p53 class mediator” (Additional file 5: Fig. S2), which also were associated with tumors. Some genes such as *Npm1*, *Dynll1*, *Ran*, *Cfl1*, *Eif5a*, *Mif*, and *Arpc2* were relative to HCC [21, 23, 25, 27–30].

### Identification of switching genes during HSC activation

**Fig. 5:**
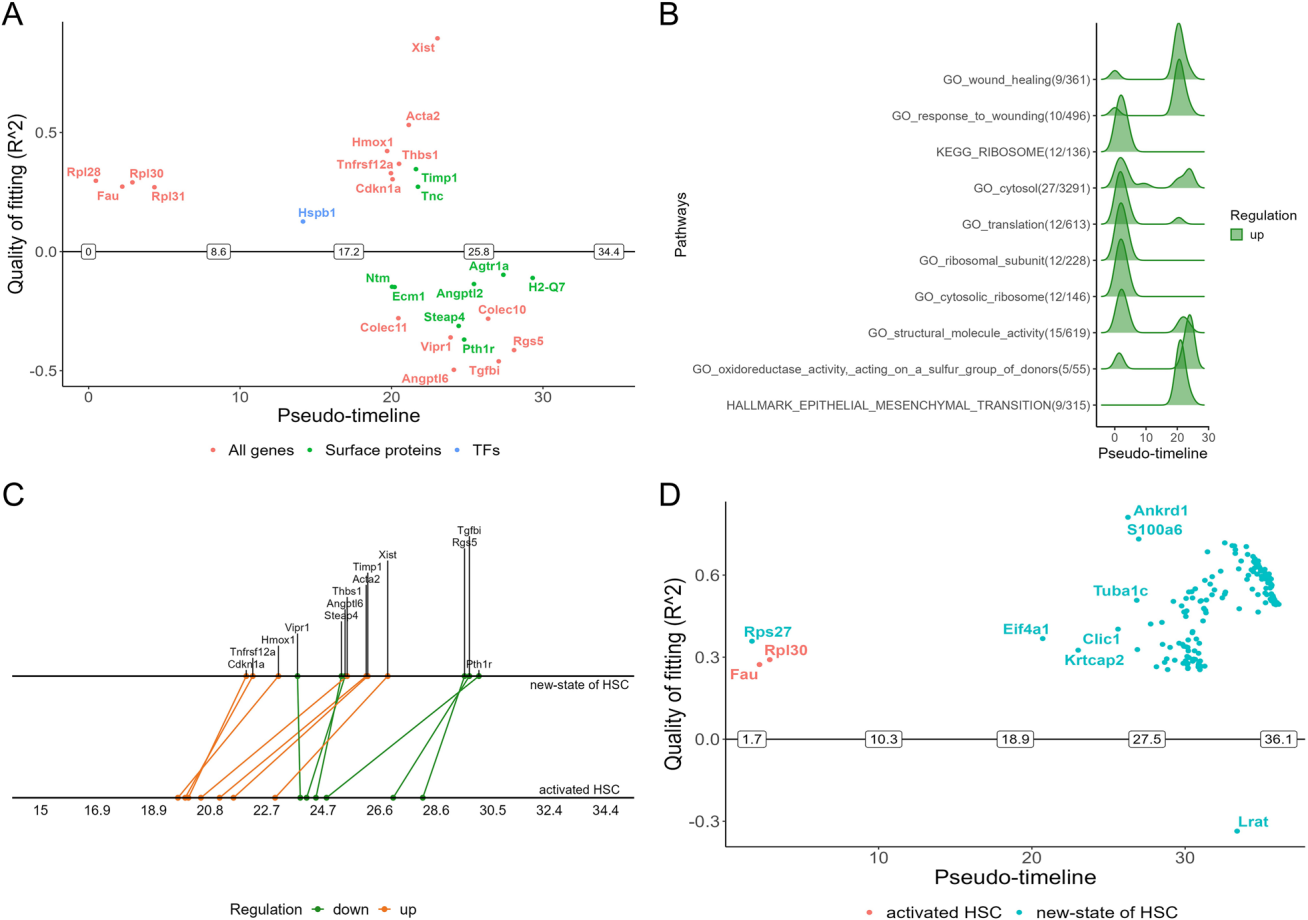
GeneSwitches analysis of scRNA-seq data from HSCs. **A** Visualization of the order of top fitting switching genes from various sets of known proteins along the pseudotime. The absolute value of the y-axis is the quality of fitting defined by McFadden's Pseudo R^2^, and the positive and negative signs indicate up- and down-regulation respectively. TFs, transcription factors. **B** Top 10 significantly changed pathways are plotted and ordered by the switching time. Ridge plots of pathways genes showing the density of switching genes. Numbers in parenthesis indicate the number of switching genes and total genes in the functional ontology respectively. **C** Visualization of the order of top fitting common switching genes between two paths along the pseudotime. **D** Visualization of top fitting distinct switching genes from the two paths along the pseudotime, i.e., genes that are only switching in one path and not the other.

In Fig. 2, GeneSwitches was first applied to the single trajectory branch 2 (state 1 and 3) in which cells differentiate from quiescent HSCs (state 1) to classic culture-activated HSCs (state 3). As shown in Fig. 5A, induction of ribosomal subunit genes was an early switching event along the pseudotime trajectory, followed by the upregulation of *Hspb1*, *Hmox1*, *Tnfrsf12a*, *Cdkn1a*, *Thbs1*, *Acta2*, *Xist*, and the downregulation of *Ntm*, *Ecm1*, *Colec11*, *Vipr1*, and *Angptl6*, etc. Among them, *Xist* (x inactive specific transcript) and *Angptl6* (angiopoietin like 6) showing the highest quality of fit determined by McFadden’s Pseudo *R*^2^ [10], may be critical switching genes to regulate HSC activation (Fig. 5A). The top biological pathways from GO and HALLMARK enrichment analysis of differentially expressed genes along the pseudotime trajectory branch 2 showed that wound healing-related genes were upregulated at an early time, followed by ribosome pathways, oxidoreductase-related ontologies, and EMT (epithelial mesenchymal transition) later in the pseudotime (Fig. 5B), simulating an in vitro gene expression pattern with similarities to that of quiescent HSC transition to myofibroblast during liver fibrogenesis.

According to the pseudotime analysis performed by Monocle2, the trajectory of HSC-to-myofibroblast transition starts from state 1, which are quiescent HSCs. Classic culture-activated HSCs are in state 3 and the novel state of HSC is state 2 (Fig. 2A). By GeneSwitches, we plotted common switching genes between two branches to compare their ordering. It showed that the top fitting common switching genes, such as *Cdkn1a*, *Tnfrsf12a*, *Hmox1*, *Thbs1*, *Acta2*, *Timp1*, and *Xist* were upregulated successively, while *Vipr1*, *Steap4*, *Angptl6*, *Rgs5*, *Tgfbi*, and *Pth1r* were downregulated (Fig. 5C). GeneSwitches also identified some distinct switching genes for the new state of HSC, such as *Rps27*, *Eif4a1*, *Krtcap2*, *Clic1*, and *Ankrd1*, etc., whereas state 3 gained only *Fau* and *Rpl30* at an early time (Fig. 5D).

### Identification of highly predictive fibrosis markers

As revealed in Fig. 2, branch 2 (state 1 and 3) follows classic HSC culture-activation trajectory, top switching genes in state 3 are potential diagnostic markers for advanced or severe fibrosis where current clinical indicators generally exhibit suboptimal sensitivity. To identify which genes are able to discriminate different stages of fibrosis, we analyzed publicly microarray data of liver biopsies from patients with NAFLD [14].

**Fig. 6:**
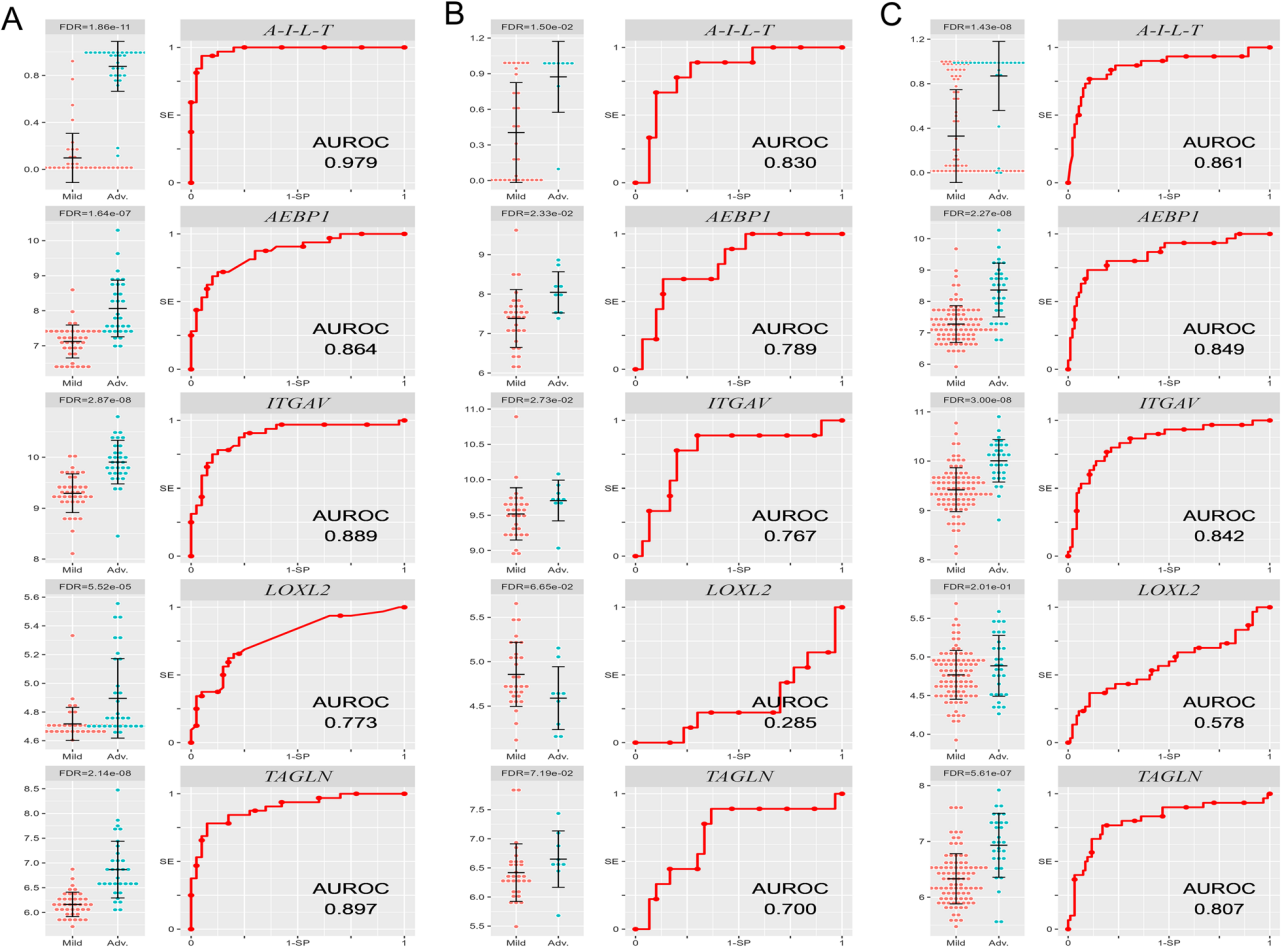
Identification of highly predictive fibrosis markers in patients with chronic liver diseases. **A** Differential expression of switching genes derived from HSC differentiation trajectory analysis in human biopsies from NAFLD patients with mild (F0–F1, n = 40) or advanced (F3–F4, n = 32) fibrosis. Average expression, standard error, and FDR-values from differential expression analysis are shown. ROC curves show the discriminatory power of each transcript and of the combination of *AEBP1*, *ITGAV*, *LOXL2*, and *TAGLN* (*A-I-L-T*). For validation of this four-gene combination, ROC curves were drawn using the microarray data from another NAFLD cohort with mild (F0–F2, n = 31) or significant (F3–F4, n = 8) fibrosis (**B**), and from HBV patients with mild (F0–F2, n = 96) or severe (F3–F4, n = 28) fibrosis (**C**). *SE* sensitivity, *SP* specificity, *AEBP1* adipocyte enhancer binding protein 1, *ITGAV* integrin subunit alpha V, *LOXL2* lysyl oxidase like 2, *TAGLN* transgelin

As shown in Fig. 6, the expression of *AEBP1*, *ITGAV*, *LOXL2*, and *TAGLN* was significantly different (false discovery rate [FDR] < 0.05) between patients with mild NAFLD (fibrosis stage F0-F1, n = 40) and advanced NAFLD (F3–F4, n = 32) (Fig. 6A). We then evaluated the diagnostic accuracy of the individual genes and a combination of four genes, *AEBP1*, *ITGAV*, *LOXL2*, and *TAGLN* (*A-I-L–T*), identified from the top switching genes by logistic regression. The model formula was shown as: $\mathrm{P }(\mathrm{probability})=\frac{{e}^{\mathrm{R}}}{1+{e}^{\mathrm{R}}}$, R = − 86.283 + (4.733 * expression level of *AEBP1*) + (5.405 * expression level of *ITGAV*) + (− 7.488 * expression level of *LOXL2*) + (5.354 * expression level of *TAGLN*). Noticeably, the combination gene *A-I-L-T* effectively identified severe fibrosis with an AUROC of 0.979 (95% confidence interval, 0.953–1.000) (Fig. 6A), sensitivity of 0.938, and specificity of 0.950, resulting in 95% detection of patients with advanced fibrosis.

Furthermore, the validation of this four-gene combination on another NAFLD cohort [15] resulted in an AUROC of 0.830 (0.689–0.971) for the identification of advanced fibrosis (stage F3–4) (Fig. 6B). In addition, on an HBV cohort [16], the *A-I-L-T* gene combination also exhibited a good discrimination for significant fibrosis (stage F3–F4) with an AUROC of 0.861 (0.772–0.951) (Fig. 6C). These findings indicated that our four-gene combination derived from HSC activation trajectory translated into human hepatic fibrosis and accurately predicted the severity of liver fibrosis caused by diverse etiology.

Most HCCs develop from severe liver fibrosis and cirrhosis. So, we investigated the mRNA expression of these four genes in HCC and normal tissues from public datasets. The results showed that *ITGAV* and *LOXL2* presented higher expression in tumor tissues than those in paracancerous tissues (TCGA-LIHC, https://portal.gdc.cancer.gov/projects/TCGA-LIHC) (*p* < 0.001, Additional file 6: Fig. S3A). A similar result was obtained in another HCC and paracancerous samples from GEO database (GSE41804) [31] (*p* < 0.05, Additional file 6: Fig. S3B).

### Validation of the genes expressed in human-derived HSC

To validate above findings in human HSC, the expression of genes relative to HSC activation, new state of HSC, and predicting fibrosis respectively were investigated between paired (quiescent and culture-activated) HSCs isolated from three human livers based on the dataset GSE68000 [32]. As revealed in Additional file 7: Fig. S4A, the expression of activation markers *ACTA2*, *MMP10*, *COL1A1*, *COL4A1*, and *COL5A2* in activated HSCs were higher than those in quiescent HSCs (*p* < 0.05). While the expression of quiescence markers *LRAT*, *RELN*, and *RGS5* was not decreased in activated HSCs. Two genes (*RACK1* and *ARPC2*) relative to the new state of HSC were expressed higher in activated HSCs than those in quiescent HSCs (*p* < 0.05, Additional file 7: Fig. S4B). The expression of other new-state related genes, such as *NPM1*, *MIF*, *KRTCAP2*, *EEF1B2*, *MRPL52*, and *SEC61B* was also increased in activated HSCs, however the differences between two groups were not statistically significant. The fibrosis marker *ITGAV* presented higher expression in activated HSCs than that in quiescent HSCs (*p* < 0.001, Additional file 7: Fig. S4C). The expression of *AEBP1*, *LOXL2*, and *TAGLN* was also increased in activated HSCs, but the differences between two groups had no statistical significance.

Recombinant human TGF-β1 can evidently enhance the activation of LX2 cells (a human HSC cell line) [33]. Then, we validated the induction of *ITGAV* and *TAGLN* by immunofluorescent analysis in LX2 cells, showing highly increased expression of both genes in TGF-β1-treated LX2 compared to untreated LX2 (Additional file 8: Fig.  S5).

## Discussion

Abnormal activation, proliferation, and migration of HSCs cause hepatic fibrosis and cirrhosis [34]. HSC-to-myofibroblast transition is a key event during liver fibrogenesis induced by diverse chronic liver injury. The scRNA-seq data (GSE132662) obtained from the HSCs cultured in vitro and harvested at day 0, 1, 3, 7, and 9 were analyzed in this study. Our results revealed a novel cell state during HSC activation, which may be relevant to CAFs. Moreover, we identified some switching genes contributing to HSC activation, and established a four-gene combination to predict advance hepatic fibrosis in patients with NAFLD or HBV.

That primary HSCs isolated from healthy mouse were cultivated for up to 7 days on uncoated plastic dishes is the classic model of HSC-to-myofibroblast transition [35]. In the present study, we in silico reconstructed a pseudotime trajectory of HSC transdifferentiation based on the single-cell transcriptomic data of the culture-activated HSC in vitro, and identified a novel state of HSC. Compared with classic activated HSC, the cells belonging to the new state express high HSC activation markers as well as relatively low collagen genes, but express high levels of genes enriched for the regulation of signal transduction by p53 class mediator, some of which were involved in the biological pathways related to cancer. For example, it has been demonstrated that *Rack1* is highly expressed by activated HSC and upregulated in HCC [36], moreover, *Rack1* can promote self-renewal of cancer stem cells in patients with HCC [22]. *Npm1* was identified in diverse cellular processes such as ribosome biogenesis, cell proliferation, and regulation of tumor suppressors p53/TP53 [37]. It’s associated with HCC [21]. The expression of *Mif* and *Cstb* in liver tissue were also verified to be involved in liver carcinogenesis [23, 24].

Liver fibrosis is a wound healing and scar repair response to liver injury. Tumors are considered as “wounds that never heal”, and more than 80% of HCC develop from cirrhosis caused by chronic liver diseases such as viral hepatitis and NAFLD [38, 39]. Activated HSCs had been found in the stroma of HCC [40], and activated peritumoral HSCs were demonstrated to be associated with tumor recurrence and mortality [41]. CAFs, a key player in hepatocarcinogenesis, probably mainly originate from HSC and may play an essential role in the pathogenesis of HCC and CCA [17, 42, 43]. TME is defined as the tumor cell population in a complex mixture of surrounding stroma, including CAFs, endothelial cells, immune cells, and ECM [44]. Recently, it has been proposed that the premalignant microenvironment (PME) should be differentiated between the TME in HCC. PME characterized by chronic liver injury, inflammation, and fibrosis, precedes tumor development [43]. We supposed that the HSCs belonging to the novel state may be an important component in PME and have the potential to convert into CAFs in TME. Further studies on the fate tracing of this specific state of HSC and regulation mechanisms of HSC-to-CAF transition may contribute to approach the goal of targeting myofibroblasts in the PME or TME for tumor prevention or therapy.

By GeneSwitches, we identified a lot of switching genes along the pseudotime trajectory of HSC transdifferentiation. *Xist*, a long noncoding RNA, can regulate HSC activation by enhancing ethanol-induced HSC autophagy [45]. Interestingly, other switching genes *Hmox1*, *Hspb1*, *Tnfrsf12a*, *Cdkn1a*, and *Thbs1* also have been regarded as modulators of autophagy [46–50]. We assumed that autophagy in HSC may strongly contribute to HSC activation at an early stage. On the other hand, the expression of *Angptl6*, a regulator of the chemotactic activity of endothelial cells and inducer of neovascularization [51] were downregulated during HSC activation. We inferred that *Angptl6* may be related to HSC functionality in healthy liver and HSC-to-myofibroblast transition in liver injury. Moreover, GeneSwitches identified some distinct switching genes for the new state of HSC and the classic culture-activated HSC, respectively. The identification of these switching genes that are specific to each trajectory (state) along with their timings may facilitate further experiments to reveal the determinants of these bifurcations.

Using Monocle2 and GeneSwitches approaches on the HSC population resolved in pseudotime, we identified top switching genes correlated with HSC activation. Our results showed that genes mirroring HSC transdifferentiation trajectory have potential diagnostic value in the prediction of advanced hepatic fibrosis from biopsy gene expression data in patients with NAFLD or HBV. Particularly, the four-gene combination, *AEBP1*, *ITGAV*, *LOXL2*, and *TAGLN*, exhibited high accuracy in predicting fibrosis severity. *AEBP1* encoded protein plays a role in adipogenesis and smooth muscle cell differentiation. Importantly, its expression upregulates with worsening fibrosis in liver biopsies from patients with nonalcoholic steatohepatitis [52]. *LOXL2* is essential to the biogenesis of connective tissue and mediates collagen crosslinking. It was strongly expressed in fibrotic liver in mice, moreover, inhibition of *LOXL2* attenuates thioacetamide-induced hepatic fibrosis [53]. Integrin αV, encoded by *ITGAV* gene, is closely associated with fibrosis in several organs [54]. *TAGLN* is an early marker of smooth muscle differentiation and regarded as an important target in anti-HBV-positive HCC [55]. According to COMPARTMENTS Subcellular localization database (http://compartments.jensenlab.org) [56], we found that *AEBP1* and *LOXL2* encode secreted proteins while *ITGAV* and *TAGLN* are located in cytosol or extracellular. Our findings indicated that *ITGAV* and *TAGLN* expression were mainly in the cytoplasm of activated human HSC. These four genes have the potential to serve as predictive markers of advanced fibrosis in patients.

There are several limitations in this study. First, the results analyzed using bulk RNA-seq data of HSCs isolated from human liver showed to some extent differences from the findings obtained from scRNA-seq data of HSCs isolated from mouse liver. Besides species difference, that the sample size of HSCs isolated from human is too small may contributed to the differences in results. In the further study, we plan to obtain scRNA-seq data of HSCs isolated from human liver including quiescent and culture-activated HSC and perform analyses to validate our findings derived from mouse HSCs. Second, before using the four-gene combination (*A-I-L-T*) to identify advanced liver fibrosis among patients with chronic liver diseases, the serum levels of transcripts or proteins of these four genes want to be evaluated in healthy and patients.

## Conclusions

In summary, we revealed a novel cell state during HSC activation and inferred that HSCs in this state may be an important origin of CAFs in TME. Moreover, we identified some critical switching genes relevant to HSC activation and established a four-gene combination which may serve as predictive markers of advanced fibrosis in patients with chronic liver diseases.

## Supplementary Information

**Additional file 1:** Supplementary methods.

**Additional file 2: Table S1**. Top 250 differentially expressed genes between two cell fate branches during HSC transdifferentiation.

**Additional file 3: Table S2**. ClueGO result table with all analysis details for the top genes in cluster 3 of Fig. 2D.

**Additional file 4: Fig. S1**. Simulation of the differentiation trajectory of HSCs isolated from healthy and CCl4-treated mouse liver and the analysis of gene expression pattern.

**Additional file 5: Fig. S2**. ClueGO and CluePedia were used for biological process annotation of the genes in cluster 3 of the branched heatmap (Fig. S1D) for HSC differentiation trajectory.

**Additional file 6: Fig. S3**. Comparison of the predictive fibrosis markers expressed in HCC and normal tissues.

**Additional file 7: Fig. S4**. Comparison of the genes expressed between paired (quiescent and culture-activated) HSCs isolated from three human livers.

**Additional file 8: Fig. S5**. Experimental verification of the expression of TAGLN and ITGAV in human hepatic stellate LX2 cells.

## Data Availability

All data analyzed during the current study are included in this article.

## Abbreviations

ECM: Extracellular matrix
HSC: Hepatic stellate cell
*Acta2*: Smooth muscle actin alpha 2
scRNA-seq: Single-cell RNA sequencing
CAFs: Cancer-associated fibroblasts
GEO: Gene Expression Omnibus
PCA: Principal component analysis
t-SNE: T-distributed stochastic neighbor embedding
MSigDB: Molecular Signatures Database
KEGG: Kyoto Encyclopedia of Genes and Genomes
NAFLD: Nonalcoholic fatty liver disease
HBV: Hepatitis B
BDL: Bile duct-ligated
DDC: 3,5-Diethoxycarbonyl-1,4-dihydrocollidine
CCA: Cholangiocarcinoma
AUROC: Area under the receiver operating characteristic curve
HCC: Hepatocellular carcinoma
*Xist*: X inactive specific transcript
*Angptl6*: Angiopoietin like 6
GO: Gene Ontology
TME: Tumor microenvironment
EMT: Epithelial mesenchymal transition
FDR: False discovery rate
PME: Premalignant microenvironment
*AEBP1*: Adipocyte enhancer binding protein 1
*ITGAV*: Integrin subunit alpha V
*LOXL2*: Lysyl oxidase like 2
*TAGLN*: Transgelin
