# Supplementary material for: Single-cell transcriptomic analysis reveals a novel cell state and switching genes during hepatic stellate cell activation in vitro
Source: J Transl Med. 2022 Jan 29;20:53. doi: 10.1186/s12967-022-03263-4 (PMC8800312; doi:10.1186/s12967-022-03263-4)
Supplement: Supplementary file 1 — Additional file 1: Supplementary methods. [file 12967_2022_3263_MOESM1_ESM.docx]

**Supplementary methods**

***Development and validation of the prediction model***

The R package Oligo (ver. 1.58.0) was used to preprocess the Affymetrix microarray data (GSE49541 and GSE84044). The R package beadarray (ver. 2.44.0) was used to preprocess the Illumina data (GSE89632). The raw data of GSE49541, GSE84044, and GSE89632 were normalized with the Robust Multi-Array Average algorithm [20]. Then, the combat function of the R language sva packages was applied to eliminate batch effects [58].

To build the prediction model based on dataset GSE49541, we firstly investigated the expression of top switching genes derived from mice between patients with mild NAFLD and advanced NAFLD. The genes with significantly different (false discovery rate < 0.05) between two groups were regarded as candidate genes. We next evaluated the diagnostic accuracy of each candidate gene based on the area under the receiver operating characteristic curve (AUROC) to identify sever fibrosis between mild NAFLD and advanced NAFLD. The genes with AUROC > 0.5 were selected for logistic regression analysis [20]. Logistic models were performed for possible combinations allowing us to calculate the probability that the expression of each combination correctly predicts fibrosis classifications. AUROC was used as an index to identify the best combination of multiple markers. At last, a minimal combination of four genes was identified among the selected individual genes by logistic regression. 

In internal validation, we applied Hosmer-Lemeshow test for the goodness-of-fit of the model and performed 1000-times-bootstrapping resampling [59] for internal calibration. Internal discrimination was assessed by the median of the AUROC for the logistic model through 1000-times-repeated bootstrapping resampling. In external validation, the same four-gene model was verified in the two validation datasets (GSE89632 and GSE84044) to calculate the probability for predicting fibrosis classifications. AUROC for the individual genes and combination were calculated using the R package pROC [18].

**References**

1. Leek JT, Johnson WE, Parker HS, Jaffe AE, Storey JD. The sva package for removing batch effects and other unwanted variation in high-throughput experiments. Bioinformatics. 2012;28(6):882-3.
2. Frank E, Harrell J. rms: Regression Modeling Strategies. R package version 5.1-4. <https://CRAN.R-project.org/package=rms>. 2019.


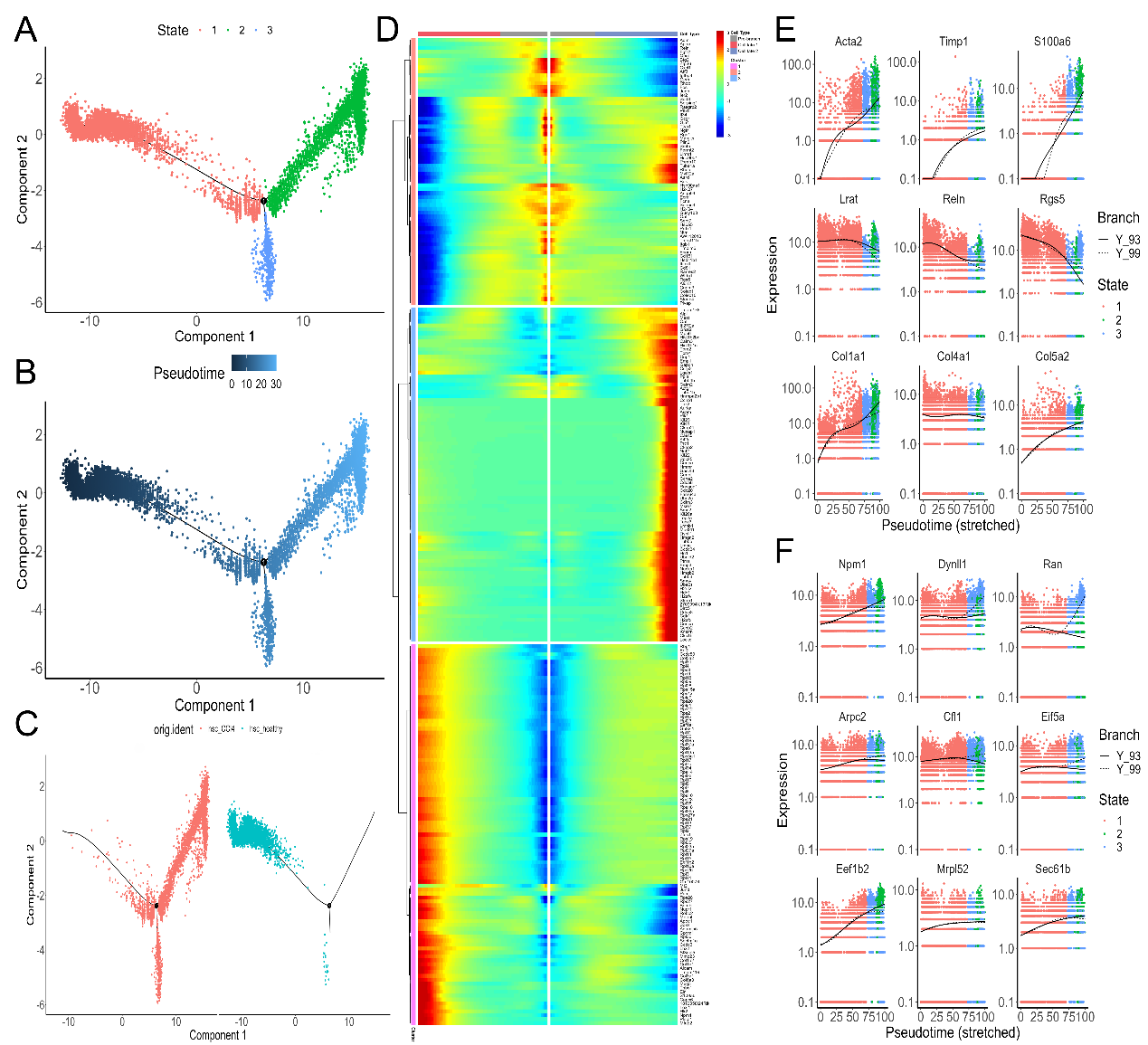


**Fig. S1** Simulation of the differentiation trajectory of HSCs isolated from healthy and CCl_4_-treated mouse liver and the analysis of gene expression pattern. (A) Trajectory reconstruction of all single cells reveals two branches. HSCs are colored by states. (B) HSCs are colored by pseudotime, showing cells developing from state 1 to the bifurcation point that gives rise to the final state 2 and 3 respectively. (C) HSCs isolated from healthy and CCl_4_-treated mouse liver are plotted along pseudotemporal trajectories. (D) The branched heatmap shows the dynamics of top 250 differentially expressed genes between the two cell fate branches during HSC transdifferentiation. Genes (rows) are divided into three clusters and cells (columns) are ordered according to the pseudotime trajectory. (E) and (F) Expression patterns of selected genes between two cell fate branches. The full line represents branch 1 (state 1 and 2) while dotted line represents branch 2 (state 1 and 3).


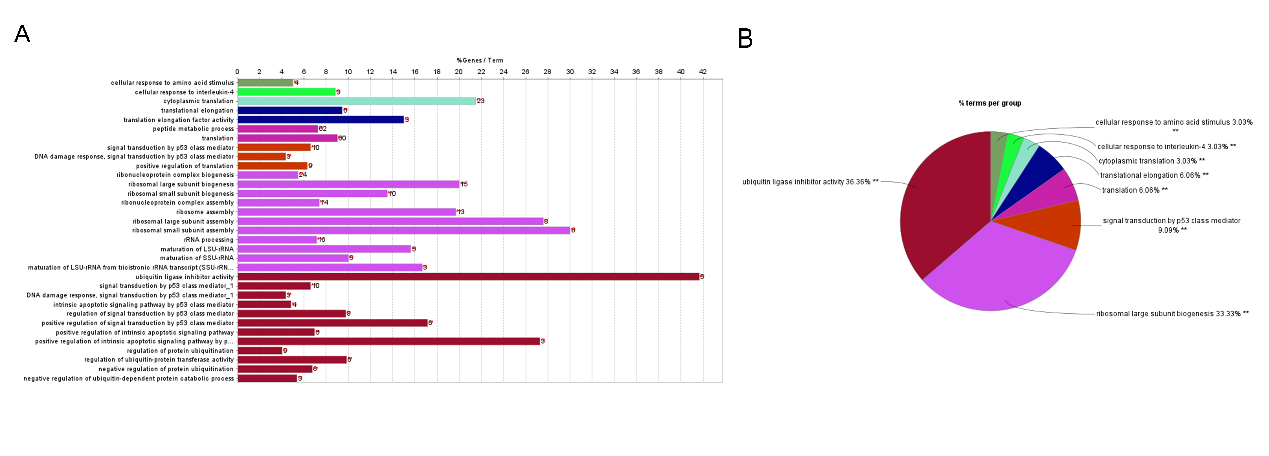


**Fig. S2** ClueGO and CluePedia were used for biological process annotation of the genes in cluster 3 of the branched heatmap (Fig. S1D) for HSC differentiation trajectory. The bar chart shows GO terms specific for the genes in cluster 3 (A). The number of genes relevant to the terms has been shown. The pie chart with functional groups shows main biological processes related to the genes in cluster 3 (B). The different groups are assigned with individual colors. The groups are ordered according to the proportion of corresponding genes belonging to the groups. Two stars indicates to P-value < 0.001.


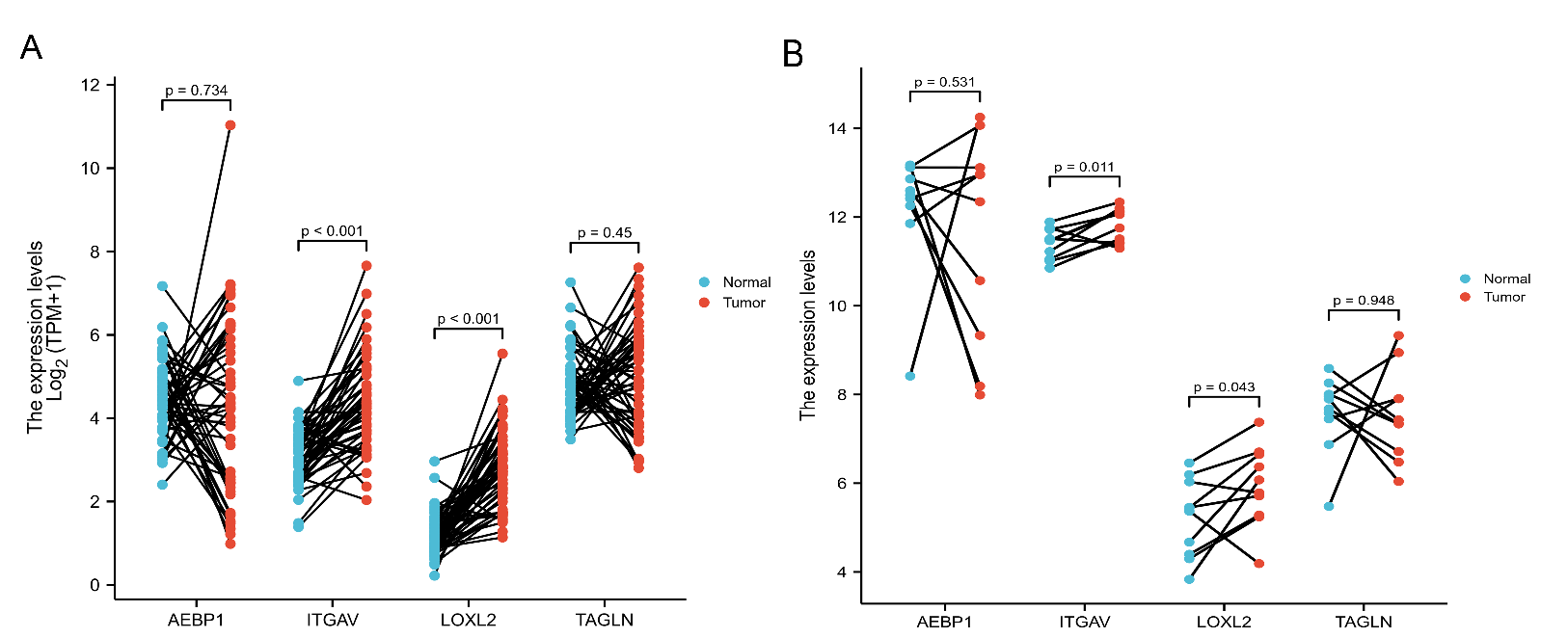


**Fig. S3** Comparison of the predictive fibrosis markers expressed in HCC and normal tissues. (A) Analysis of mRNA expression of *AEBP1*, *ITGAV*, *LOXL2*, and *TAGLN* in 50 HCC and paracancerous tissues based on TCGA-LIHC (https://portal.gdc.cancer.gov/projects/TCGA-LIHC). (B) Expression of *AEBP1*, *ITGAV*, *LOXL2*, and *TAGLN* mRNA in 20 HCC and non-cancerous tissues according to GSE41804 dataset.


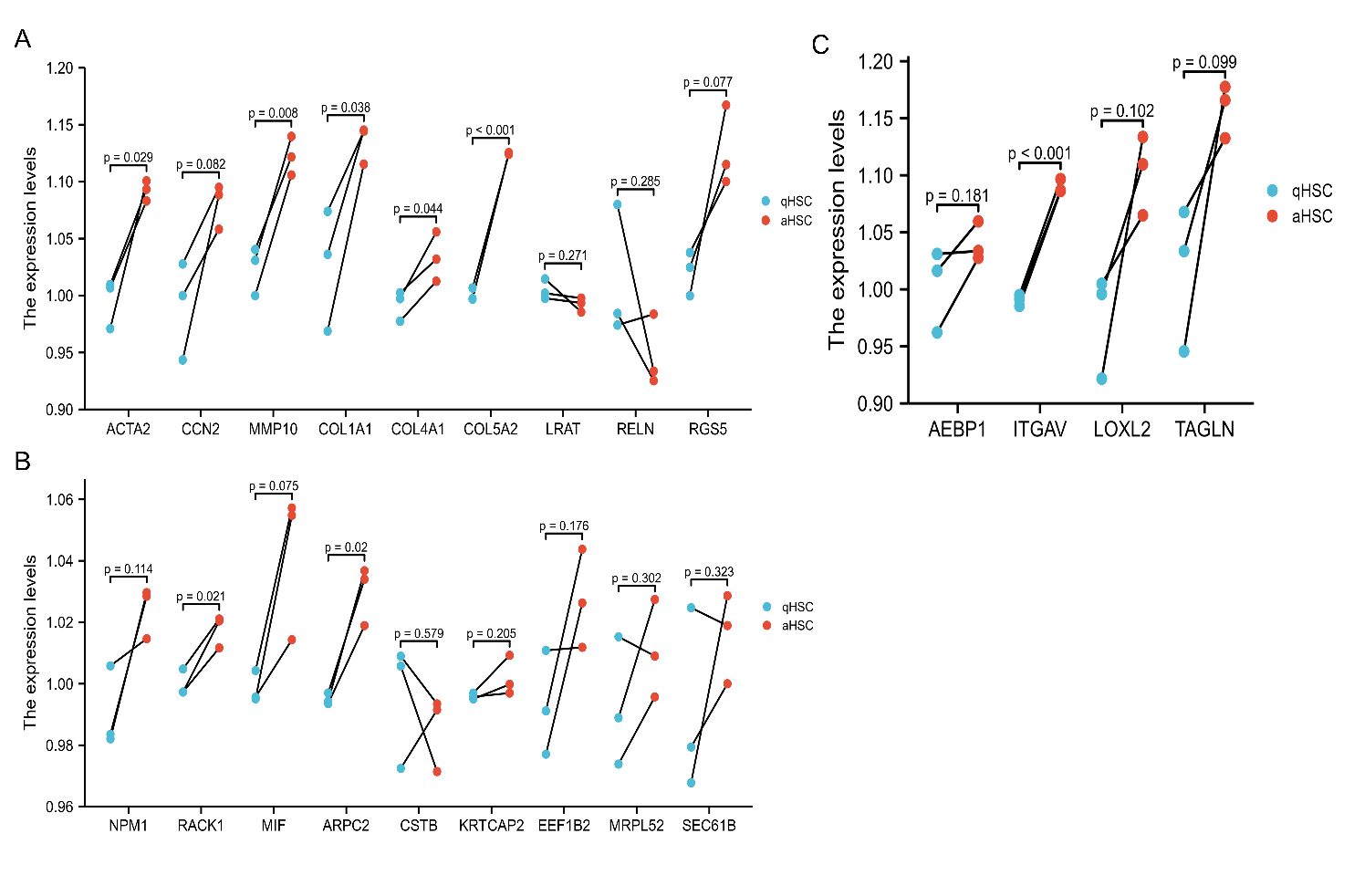


**Fig. S4** Comparison of the genes expressed between paired (quiescent and culture-activated) HSCs isolated from three human livers based on the dataset GSE68000. (A) Expression of the genes related to HSC activation. (B) Expression of the genes relative to the new state of HSC. (C) Expression of the predictive fibrosis markers. qHSC, quiescent hepatic stellate cell; aHSC, activated hepatic stellate cell.


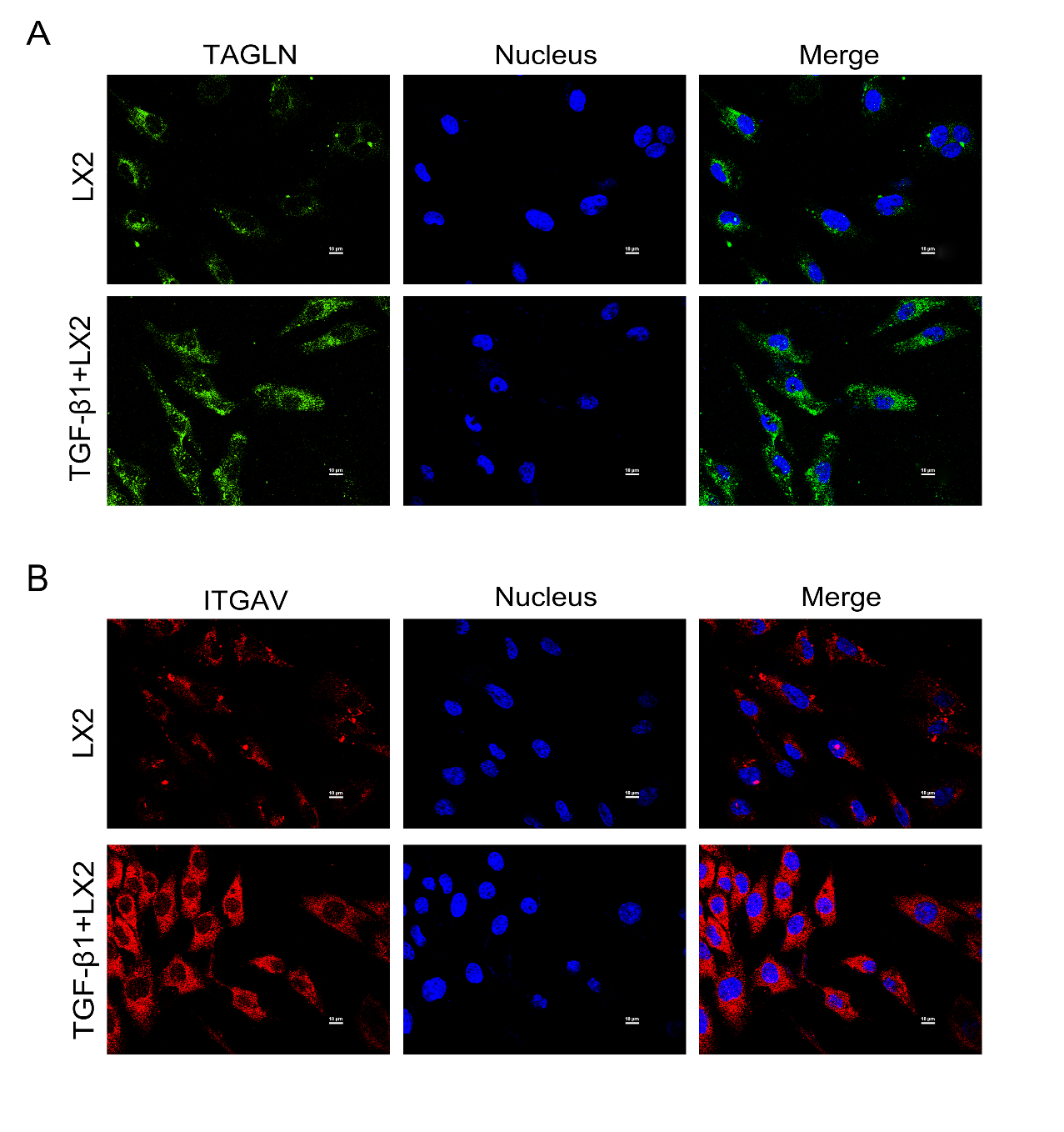


**Fig. S5** Experimental verification of the expression of *TAGLN* and *ITGAV* in human hepatic stellate LX2 cells. LX-2 were treated with recombinant human TGF-β1 (10 ng/ml) for 24 h. Immunofluorescence assay was used to demonstrate the expression of *TAGLN* (A) and *ITGAV* (B) in LX2 and TGF-β1-treated LX2. Nuclei were stained with DAPI. The bar represents 10 μm. TGF-β1, transforming growth factor β1; *ITGAV*, integrin Subunit Alpha V; *TAGLN*, transgelin.
