# Supplementary material for: Single-cell transcriptomic analysis reveals a novel cell state and switching genes during hepatic stellate cell activation in vitro
Source: J Transl Med. 2022 Jan 29;20:53. doi: 10.1186/s12967-022-03263-4 (PMC8800312; doi:10.1186/s12967-022-03263-4)
Supplement: Supplementary file 2 — Additional file 2: Table S1. Top 250 differentially expressed genes between two cell fate branches during HSC transdifferentiation. [file 12967_2022_3263_MOESM2_ESM.docx]

**Table S1.** Top 250 differentially expressed genes between two cell fate branches during HSC transdifferentiation.

| **Gene** | **pval** | **qval** | **Cluster** |
| --- | --- | --- | --- |
| Acta2 | 8.97E-51 | 8.47E-50 | 1 |
| Actn1 | 1.04E-58 | 1.17E-57 | 1 |
| Adam9 | 5.87E-55 | 5.93E-54 | 1 |
| Ahnak | 5.38E-174 | 2.44E-172 | 1 |
| Angptl4 | 1.21E-40 | 9.69E-40 | 1 |
| Axl | 2.97E-113 | 5.72E-112 | 1 |
| Bmper | 1.70E-45 | 1.49E-44 | 1 |
| Cald1 | 1.07E-51 | 1.03E-50 | 1 |
| Ccn1 | 3.56E-75 | 5.05E-74 | 1 |
| Ccn2 | 1.19E-118 | 2.38E-117 | 1 |
| Cdkn1a | 6.29E-43 | 5.31E-42 | 1 |
| Cemip | 2.95E-47 | 2.65E-46 | 1 |
| Ckb | 6.08E-58 | 6.68E-57 | 1 |
| Col12a1 | 1.96E-74 | 2.75E-73 | 1 |
| Col1a1 | 0 | 0 | 1 |
| Col4a1 | 0 | 0 | 1 |
| Col4a2 | 1.92E-291 | 2.74E-289 | 1 |
| Col5a2 | 0 | 0 | 1 |
| Col8a1 | 1.16E-67 | 1.47E-66 | 1 |
| Crct1 | 1.89E-169 | 7.87E-168 | 1 |
| Csf1 | 5.51E-126 | 1.22E-124 | 1 |
| Ctsd | 3.15E-159 | 1.15E-157 | 1 |
| Dst | 8.26E-93 | 1.33E-91 | 1 |
| Fat1 | 6.09E-74 | 8.40E-73 | 1 |
| Fbln2 | 7.38E-153 | 2.42E-151 | 1 |
| Flna | 5.28E-153 | 1.76E-151 | 1 |
| Flnb | 4.52E-50 | 4.22E-49 | 1 |
| Frzb | 2.99E-54 | 3.01E-53 | 1 |
| Hs6st2 | 2.06E-42 | 1.73E-41 | 1 |
| Hspg2 | 1.17E-219 | 1.01E-217 | 1 |
| Itgav | 3.66E-119 | 7.47E-118 | 1 |
| Large1 | 3.59E-52 | 3.50E-51 | 1 |
| Lox | 1.26E-100 | 2.17E-99 | 1 |
| Lpp | 1.15E-41 | 9.50E-41 | 1 |
| Mmp10 | 1.19E-163 | 4.65E-162 | 1 |
| mt-Atp6 | 0 | 0 | 1 |
| mt-Co1 | 0 | 0 | 1 |
| mt-Co2 | 0 | 0 | 1 |
| mt-Co3 | 0 | 0 | 1 |
| mt-Cytb | 0 | 0 | 1 |
| mt-Nd1 | 0 | 0 | 1 |
| mt-Nd4 | 0 | 0 | 1 |
| Nav2 | 1.18E-57 | 1.29E-56 | 1 |
| Neat1 | 3.45E-270 | 4.61E-268 | 1 |
| Nes | 8.51E-59 | 9.67E-58 | 1 |
| Npr3 | 3.70E-74 | 5.14E-73 | 1 |
| Pcdh7 | 2.88E-87 | 4.36E-86 | 1 |
| Peak1 | 1.24E-57 | 1.35E-56 | 1 |
| Phldb2 | 2.68E-58 | 2.97E-57 | 1 |
| Piezo1 | 2.31E-44 | 1.99E-43 | 1 |
| Plec | 1.95E-71 | 2.63E-70 | 1 |
| Prss23 | 2.71E-65 | 3.32E-64 | 1 |
| Qsox1 | 2.81E-69 | 3.67E-68 | 1 |
| Robo2 | 8.38E-52 | 8.14E-51 | 1 |
| Serpine1 | 3.91E-257 | 4.61E-255 | 1 |
| Slco2a1 | 2.69E-45 | 2.35E-44 | 1 |
| Sparc | 3.08E-134 | 7.71E-133 | 1 |
| Sprr1a | 8.58E-264 | 1.07E-261 | 1 |
| Synpo | 7.24E-79 | 1.04E-77 | 1 |
| Thbs1 | 0 | 0 | 1 |
| Tinagl1 | 3.12E-106 | 5.62E-105 | 1 |
| Tnc | 1.43E-119 | 2.96E-118 | 1 |
| Unc5b | 2.70E-123 | 5.69E-122 | 1 |
| Vcp | 2.01E-81 | 2.96E-80 | 1 |
| Xist | 0 | 0 | 1 |
| Adamts2 | 1.26E-62 | 1.51E-61 | 2 |
| App | 2.40E-93 | 3.91E-92 | 2 |
| B2m | 3.86E-57 | 4.13E-56 | 2 |
| Calr | 3.02E-66 | 3.72E-65 | 2 |
| Ccdc80 | 1.30E-68 | 1.66E-67 | 2 |
| Ccl2 | 1.90E-72 | 2.59E-71 | 2 |
| Ccnt2 | 3.71E-49 | 3.42E-48 | 2 |
| Clca3a1 | 3.70E-108 | 6.79E-107 | 2 |
| Col14a1 | 6.98E-119 | 1.41E-117 | 2 |
| Col1a2 | 3.17E-69 | 4.11E-68 | 2 |
| Col3a1 | 2.10E-100 | 3.59E-99 | 2 |
| Col6a3 | 4.99E-45 | 4.32E-44 | 2 |
| Ctsl | 1.42E-145 | 4.19E-144 | 2 |
| Cxcl12 | 6.84E-41 | 5.51E-40 | 2 |
| Dcn | 9.66E-44 | 8.29E-43 | 2 |
| Ece1 | 1.07E-59 | 1.24E-58 | 2 |
| Ecm1 | 9.24E-64 | 1.11E-62 | 2 |
| Efemp1 | 1.11E-50 | 1.04E-49 | 2 |
| Emilin1 | 3.07E-86 | 4.59E-85 | 2 |
| Eng | 1.19E-56 | 1.24E-55 | 2 |
| Fn1 | 1.73E-76 | 2.47E-75 | 2 |
| Hes1 | 3.32E-85 | 4.92E-84 | 2 |
| Hsp90b1 | 3.69E-89 | 5.77E-88 | 2 |
| Hspa5 | 7.09E-113 | 1.35E-111 | 2 |
| Il6st | 2.42E-43 | 2.05E-42 | 2 |
| Itga9 | 3.66E-42 | 3.05E-41 | 2 |
| Itpr1 | 6.92E-47 | 6.15E-46 | 2 |
| Lamb1 | 5.06E-141 | 1.37E-139 | 2 |
| Lamc1 | 1.64E-68 | 2.10E-67 | 2 |
| Lrp1 | 1.38E-215 | 1.10E-213 | 2 |
| Luc7l2 | 4.33E-59 | 4.98E-58 | 2 |
| Macf1 | 2.76E-61 | 3.24E-60 | 2 |
| Malat1 | 0 | 0 | 2 |
| Mxra8 | 6.04E-56 | 6.19E-55 | 2 |
| Nisch | 3.36E-41 | 2.72E-40 | 2 |
| Ntn1 | 9.25E-52 | 8.90E-51 | 2 |
| Ogt | 4.03E-89 | 6.25E-88 | 2 |
| Pcolce | 3.05E-129 | 7.18E-128 | 2 |
| Pla2r1 | 2.07E-46 | 1.83E-45 | 2 |
| Plvap | 8.30E-147 | 2.55E-145 | 2 |
| Postn | 1.81E-58 | 2.02E-57 | 2 |
| Prelp | 2.01E-80 | 2.91E-79 | 2 |
| Psap | 1.08E-148 | 3.39E-147 | 2 |
| Pxdn | 9.96E-90 | 1.58E-88 | 2 |
| Raph1 | 3.69E-234 | 3.69E-232 | 2 |
| Rcn3 | 3.68E-47 | 3.28E-46 | 2 |
| Reln | 1.98E-89 | 3.11E-88 | 2 |
| Rsrp1 | 1.38E-47 | 1.25E-46 | 2 |
| Serpinh1 | 3.25E-69 | 4.19E-68 | 2 |
| Son | 5.78E-99 | 9.64E-98 | 2 |
| Tcn2 | 1.91E-43 | 1.62E-42 | 2 |
| Zbtb20 | 2.22E-101 | 3.87E-100 | 2 |
| Arpc2 | 9.76E-42 | 8.06E-41 | 3 |
| Atp5e | 1.97E-51 | 1.87E-50 | 3 |
| Atp5g1 | 3.27E-61 | 3.82E-60 | 3 |
| Atp5g2 | 1.65E-56 | 1.71E-55 | 3 |
| Atp5h | 3.14E-41 | 2.56E-40 | 3 |
| Atp5l | 2.51E-41 | 2.04E-40 | 3 |
| Atp5mpl | 2.60E-51 | 2.46E-50 | 3 |
| Calm1 | 1.91E-52 | 1.90E-51 | 3 |
| Ccl7 | 6.21E-49 | 5.70E-48 | 3 |
| Cfl1 | 1.16E-108 | 2.17E-107 | 3 |
| Cox7a2 | 3.19E-52 | 3.13E-51 | 3 |
| Cox7c | 7.69E-70 | 1.03E-68 | 3 |
| Cox8a | 3.58E-47 | 3.21E-46 | 3 |
| Cstb | 8.45E-52 | 8.16E-51 | 3 |
| Dynll1 | 9.96E-70 | 1.32E-68 | 3 |
| Eef1b2 | 2.80E-66 | 3.47E-65 | 3 |
| Eif5a | 4.71E-102 | 8.33E-101 | 3 |
| Elob | 5.17E-59 | 5.91E-58 | 3 |
| Fau | 7.44E-89 | 1.14E-87 | 3 |
| Fth1 | 1.98E-235 | 2.09E-233 | 3 |
| Ftl1 | 1.35E-221 | 1.23E-219 | 3 |
| Gng5 | 2.18E-87 | 3.33E-86 | 3 |
| Hspa1a | 2.12E-128 | 4.82E-127 | 3 |
| Hspe1 | 8.93E-65 | 1.08E-63 | 3 |
| Krtcap2 | 6.71E-57 | 7.11E-56 | 3 |
| Loxl2 | 5.37E-160 | 1.99E-158 | 3 |
| Mif | 2.57E-71 | 3.45E-70 | 3 |
| Mrpl52 | 4.63E-54 | 4.63E-53 | 3 |
| Naca | 2.03E-57 | 2.19E-56 | 3 |
| Ndufa2 | 2.41E-57 | 2.59E-56 | 3 |
| Ndufa4 | 1.17E-56 | 1.23E-55 | 3 |
| Npm1 | 5.65E-67 | 7.10E-66 | 3 |
| Ppia | 1.67E-108 | 3.10E-107 | 3 |
| Rack1 | 2.30E-63 | 2.76E-62 | 3 |
| Ran | 8.74E-62 | 1.03E-60 | 3 |
| Ranbp1 | 1.03E-46 | 9.10E-46 | 3 |
| Rpl10a | 6.91E-67 | 8.63E-66 | 3 |
| Rpl11 | 1.66E-102 | 2.97E-101 | 3 |
| Rpl12 | 2.36E-153 | 8.27E-152 | 3 |
| Rpl13 | 3.92E-178 | 1.91E-176 | 3 |
| Rpl13a | 3.90E-153 | 1.32E-151 | 3 |
| Rpl14 | 3.65E-125 | 7.93E-124 | 3 |
| Rpl17 | 2.87E-202 | 1.85E-200 | 3 |
| Rpl18 | 5.25E-117 | 1.04E-115 | 3 |
| Rpl18a | 5.08E-133 | 1.26E-131 | 3 |
| Rpl19 | 2.26E-134 | 5.71E-133 | 3 |
| Rpl21 | 2.48E-152 | 7.99E-151 | 3 |
| Rpl22 | 3.40E-100 | 5.76E-99 | 3 |
| Rpl23 | 6.29E-130 | 1.50E-128 | 3 |
| Rpl23a | 2.53E-116 | 4.97E-115 | 3 |
| Rpl24 | 2.25E-137 | 5.84E-136 | 3 |
| Rpl26 | 1.32E-146 | 3.94E-145 | 3 |
| Rpl27a | 1.83E-96 | 3.00E-95 | 3 |
| Rpl28 | 3.28E-126 | 7.37E-125 | 3 |
| Rpl29 | 6.75E-139 | 1.80E-137 | 3 |
| Rpl3 | 4.71E-87 | 7.08E-86 | 3 |
| Rpl30 | 7.68E-65 | 9.37E-64 | 3 |
| Rpl31 | 2.14E-131 | 5.15E-130 | 3 |
| Rpl32 | 2.44E-171 | 1.08E-169 | 3 |
| Rpl34 | 5.27E-183 | 2.70E-181 | 3 |
| Rpl35 | 3.17E-184 | 1.71E-182 | 3 |
| Rpl35a | 1.62E-192 | 9.79E-191 | 3 |
| Rpl36 | 2.56E-161 | 9.68E-160 | 3 |
| Rpl36a | 3.36E-115 | 6.53E-114 | 3 |
| Rpl37 | 1.52E-176 | 7.25E-175 | 3 |
| Rpl37a | 2.05E-170 | 8.93E-169 | 3 |
| Rpl38 | 1.35E-165 | 5.41E-164 | 3 |
| Rpl39 | 1.37E-168 | 5.58E-167 | 3 |
| Rpl4 | 1.58E-69 | 2.08E-68 | 3 |
| Rpl41 | 4.06E-205 | 2.80E-203 | 3 |
| Rpl5 | 1.19E-56 | 1.24E-55 | 3 |
| Rpl6 | 2.30E-121 | 4.80E-120 | 3 |
| Rpl7 | 3.41E-97 | 5.64E-96 | 3 |
| Rpl7a | 1.38E-101 | 2.42E-100 | 3 |
| Rpl8 | 1.72E-107 | 3.12E-106 | 3 |
| Rplp0 | 6.59E-190 | 3.87E-188 | 3 |
| Rplp1 | 8.75E-184 | 4.61E-182 | 3 |
| Rplp2 | 6.95E-126 | 1.53E-124 | 3 |
| Rps10 | 1.95E-134 | 4.99E-133 | 3 |
| Rps11 | 2.04E-128 | 4.69E-127 | 3 |
| Rps12 | 3.53E-189 | 1.96E-187 | 3 |
| Rps13 | 4.56E-143 | 1.28E-141 | 3 |
| Rps14 | 4.89E-145 | 1.40E-143 | 3 |
| Rps15 | 2.61E-56 | 2.70E-55 | 3 |
| Rps15a | 7.23E-231 | 6.89E-229 | 3 |
| Rps16 | 1.41E-161 | 5.41E-160 | 3 |
| Rps17 | 1.42E-138 | 3.74E-137 | 3 |
| Rps18 | 1.47E-182 | 7.35E-181 | 3 |
| Rps19 | 1.81E-176 | 8.43E-175 | 3 |
| Rps2 | 1.92E-202 | 1.28E-200 | 3 |
| Rps20 | 1.29E-92 | 2.07E-91 | 3 |
| Rps23 | 8.24E-206 | 6.10E-204 | 3 |
| Rps24 | 2.48E-205 | 1.77E-203 | 3 |
| Rps25 | 1.91E-155 | 6.82E-154 | 3 |
| Rps26 | 1.49E-124 | 3.20E-123 | 3 |
| Rps27a | 1.50E-192 | 9.40E-191 | 3 |
| Rps27l | 7.01E-124 | 1.49E-122 | 3 |
| Rps28 | 1.61E-217 | 1.34E-215 | 3 |
| Rps29 | 7.47E-249 | 8.30E-247 | 3 |
| Rps3 | 4.20E-132 | 1.03E-130 | 3 |
| Rps3a1 | 3.32E-153 | 1.14E-151 | 3 |
| Rps4x | 6.16E-129 | 1.43E-127 | 3 |
| Rps5 | 2.84E-170 | 1.21E-168 | 3 |
| Rps6 | 9.95E-208 | 7.65E-206 | 3 |
| Rps7 | 3.60E-145 | 1.04E-143 | 3 |
| Rps8 | 1.28E-146 | 3.88E-145 | 3 |
| Rps9 | 1.37E-142 | 3.74E-141 | 3 |
| Rpsa | 8.99E-143 | 2.50E-141 | 3 |
| Sec61b | 5.68E-57 | 6.04E-56 | 3 |
| Sec61g | 1.81E-80 | 2.64E-79 | 3 |
| Sem1 | 3.99E-55 | 4.07E-54 | 3 |
| Serf2 | 6.96E-74 | 9.54E-73 | 3 |
| Spon2 | 6.82E-50 | 6.34E-49 | 3 |
| Srp14 | 3.16E-45 | 2.74E-44 | 3 |
| Tmsb4x | 1.36E-151 | 4.33E-150 | 3 |
| Tomm20 | 2.80E-52 | 2.76E-51 | 3 |
| Tpt1 | 2.23E-189 | 1.27E-187 | 3 |
| Txn1 | 2.39E-74 | 3.34E-73 | 3 |
| Ubc | 1.64E-58 | 1.84E-57 | 3 |
| Uqcrh | 1.47E-42 | 1.24E-41 | 3 |
| Uqcrq | 1.96E-52 | 1.94E-51 | 3 |
| Vmp1 | 1.02E-49 | 9.44E-49 | 3 |
| Cnbp | 9.03E-41 | 7.25E-40 | 4 |
| Gm10073 | 4.60E-55 | 4.68E-54 | 4 |
| Gm8730 | 3.87E-42 | 3.21E-41 | 4 |
| Gm9493 | 1.03E-43 | 8.77E-43 | 4 |
| H3f3a | 5.02E-48 | 4.58E-47 | 4 |
| Ifitm3 | 9.22E-60 | 1.07E-58 | 4 |
| Mustn1 | 3.86E-58 | 4.27E-57 | 4 |
| Ptma | 2.15E-111 | 4.06E-110 | 4 |
| Rpl23a-ps3 | 2.73E-47 | 2.47E-46 | 4 |
| Rps12-ps3 | 1.38E-99 | 2.32E-98 | 4 |
| Rps27rt | 1.35E-41 | 1.11E-40 | 4 |
