# Supplementary material for: Single-cell transcriptomic analysis reveals a novel cell state and switching genes during hepatic stellate cell activation in vitro
Source: J Transl Med. 2022 Jan 29;20:53. doi: 10.1186/s12967-022-03263-4 (PMC8800312; doi:10.1186/s12967-022-03263-4)
Supplement: Supplementary file 3 — Additional file 3: Table S2. ClueGO result table with all analysis details for the top genes in cluster 3 of Fig. 2D. [file 12967_2022_3263_MOESM3_ESM.docx]

**Table S2.** ClueGO result table with all analysis details for the top genes in cluster 3 of Figure 2D.

| **ID** | **Term** | **% Associated Genes** | **GOGroups** | **Nr. Genes** | **Associated Genes Found** |
| --- | --- | --- | --- | --- | --- |
| GO:0006414 | translational elongation | 9.43 | Group00 | 5 | [Eef1b2, Eif5a, Rack1, Rplp1, Rplp2] |
| GO:0071353 | cellular response to interleukin-4 | 8.82 | Group01 | 3 | [Rpl3, Rplp0, Rps2] |
| GO:0002181 | cytoplasmic translation | 27.1 | Group02 | 29 | [Rpl10a, Rpl11, Rpl13a, Rpl17, Rpl18, Rpl18a, Rpl19, Rpl22, Rpl24, Rpl26, Rpl29, Rpl30, Rpl31, Rpl32, Rpl35a, Rpl36, Rpl38, Rpl39, Rpl6, Rpl8, Rplp0, Rplp1, Rplp2, Rps2, Rps23, Rps26, Rps28, Rps29, Rpsa] |
| GO:0065002 | intracellular protein transmembrane transport | 5.17 | Group03 | 3 | [Sec61b, Sec61g, Tomm20] |
| GO:0045047 | protein targeting to ER | 6.82 | Group03 | 3 | [Sec61b, Sec61g, Srp14] |
| GO:1901566 | organonitrogen compound biosynthetic process | 5.09 | Group04 | 82 | [Atp5e, Atp5g1, Atp5g2, Atp5h, Atp5l, Eef1b2, Eif5a, Fau, Krtcap2, Mrpl52, Npm1, Rack1, Rpl10a, Rpl11, Rpl12, Rpl13, Rpl13a, Rpl14, Rpl17, Rpl18, Rpl18a, Rpl19, Rpl21, Rpl22, Rpl23, Rpl23a, Rpl24, Rpl26, Rpl27a, Rpl28, Rpl29, Rpl3, Rpl30, Rpl31, Rpl32, Rpl34, Rpl35, Rpl35a, Rpl36, Rpl36a, Rpl37, Rpl37a, Rpl38, Rpl39, Rpl4, Rpl41, Rpl5, Rpl6, Rpl7, Rpl8, Rplp0, Rplp1, Rplp2, Rps11, Rps12, Rps13, Rps14, Rps15, Rps15a, Rps16, Rps17, Rps18, Rps19, Rps2, Rps20, Rps23, Rps24, Rps26, Rps27a, Rps27l, Rps28, Rps29, Rps3, Rps3a1, Rps4x, Rps5, Rps6, Rps7, Rps8, Rps9, Rpsa, Ubc] |
| GO:0006412 | translation | 11.41 | Group04 | 76 | [Eef1b2, Eif5a, Fau, Mrpl52, Npm1, Rack1, Rpl10a, Rpl11, Rpl12, Rpl13, Rpl13a, Rpl14, Rpl17, Rpl18, Rpl18a, Rpl19, Rpl21, Rpl22, Rpl23, Rpl23a, Rpl24, Rpl26, Rpl27a, Rpl28, Rpl29, Rpl3, Rpl30, Rpl31, Rpl32, Rpl34, Rpl35, Rpl35a, Rpl36, Rpl36a, Rpl37, Rpl37a, Rpl38, Rpl39, Rpl4, Rpl41, Rpl5, Rpl6, Rpl7, Rpl8, Rplp0, Rplp1, Rplp2, Rps11, Rps12, Rps13, Rps14, Rps15, Rps15a, Rps16, Rps17, Rps18, Rps19, Rps2, Rps20, Rps23, Rps24, Rps26, Rps27a, Rps27l, Rps28, Rps29, Rps3, Rps3a1, Rps4x, Rps5, Rps6, Rps7, Rps8, Rps9, Rpsa, Ubc] |
| GO:0042273 | ribosomal large subunit biogenesis | 21.33 | Group05 | 16 | [Npm1, Rpl10a, Rpl11, Rpl14, Rpl23a, Rpl24, Rpl26, Rpl3, Rpl35, Rpl35a, Rpl38, Rpl5, Rpl6, Rpl7, Rpl7a, Rplp0] |
| GO:0000470 | maturation of LSU-rRNA | 18.75 | Group05 | 6 | [Npm1, Rpl10a, Rpl35, Rpl35a, Rpl7, Rpl7a] |
| GO:0000463 | maturation of LSU-rRNA from tricistronic rRNA transcript (SSU-rRNA, 5.8S rRNA, LSU-rRNA) | 22.22 | Group05 | 4 | [Npm1, Rpl35, Rpl35a, Rpl7] |
| GO:0071426 | ribonucleoprotein complex export from nucleus | 6.94 | Group06 | 5 | [Eif5a, Npm1, Ran, Rps15, Sem1] |
| GO:0097421 | liver regeneration | 8.89 | Group06 | 4 | [Npm1, Rpl19, Rps15, Rps16] |
| GO:0000056 | ribosomal small subunit export from nucleus | 30 | Group06 | 3 | [Npm1, Ran, Rps15] |
| GO:0022613 | ribonucleoprotein complex biogenesis | 7.5 | Group07 | 33 | [Npm1, Ran, Rpl10a, Rpl11, Rpl13a, Rpl14, Rpl23a, Rpl24, Rpl26, Rpl3, Rpl35, Rpl35a, Rpl38, Rpl5, Rpl6, Rpl7, Rpl7a, Rplp0, Rps14, Rps15, Rps16, Rps17, Rps19, Rps2, Rps24, Rps25, Rps27l, Rps28, Rps5, Rps6, Rps7, Rps8, Rpsa] |
| GO:0042273 | ribosomal large subunit biogenesis | 21.33 | Group07 | 16 | [Npm1, Rpl10a, Rpl11, Rpl14, Rpl23a, Rpl24, Rpl26, Rpl3, Rpl35, Rpl35a, Rpl38, Rpl5, Rpl6, Rpl7, Rpl7a, Rplp0] |
| GO:0042274 | ribosomal small subunit biogenesis | 22.97 | Group07 | 17 | [Npm1, Rpl38, Rps14, Rps15, Rps16, Rps17, Rps19, Rps2, Rps24, Rps25, Rps27l, Rps28, Rps5, Rps6, Rps7, Rps8, Rpsa] |
| GO:0022618 | ribonucleoprotein complex assembly | 9.47 | Group07 | 18 | [Rpl11, Rpl13a, Rpl23a, Rpl24, Rpl3, Rpl38, Rpl5, Rpl6, Rplp0, Rps14, Rps15, Rps19, Rps2, Rps25, Rps27l, Rps28, Rps5, Rpsa] |
| GO:0042255 | ribosome assembly | 25.76 | Group07 | 17 | [Rpl11, Rpl23a, Rpl24, Rpl3, Rpl38, Rpl5, Rpl6, Rplp0, Rps14, Rps15, Rps19, Rps2, Rps25, Rps27l, Rps28, Rps5, Rpsa] |
| GO:0000027 | ribosomal large subunit assembly | 27.59 | Group07 | 8 | [Rpl11, Rpl23a, Rpl24, Rpl3, Rpl38, Rpl5, Rpl6, Rplp0] |
| GO:0000028 | ribosomal small subunit assembly | 50 | Group07 | 10 | [Rpl38, Rps14, Rps15, Rps19, Rps2, Rps25, Rps27l, Rps28, Rps5, Rpsa] |
| GO:0006364 | rRNA processing | 9.38 | Group07 | 21 | [Npm1, Rpl10a, Rpl11, Rpl14, Rpl26, Rpl35, Rpl35a, Rpl5, Rpl7, Rpl7a, Rps14, Rps15, Rps16, Rps17, Rps19, Rps24, Rps25, Rps28, Rps6, Rps7, Rps8] |
| GO:0000470 | maturation of LSU-rRNA | 18.75 | Group07 | 6 | [Npm1, Rpl10a, Rpl35, Rpl35a, Rpl7, Rpl7a] |
| GO:0030490 | maturation of SSU-rRNA | 10 | Group07 | 5 | [Rps14, Rps16, Rps19, Rps28, Rps8] |
| GO:1990542 | mitochondrial transmembrane transport | 4.17 | Group08 | 4 | [Atp5e, Atp5h, Atp5l, Tomm20] |
| GO:0006119 | oxidative phosphorylation | 7.02 | Group08 | 8 | [Atp5e, Atp5h, Atp5l, Cox7a2, Cox7c, Cox8a, Uqcrh, Uqcrq] |
| GO:0009055 | electron transfer activity | 8.24 | Group08 | 7 | [Cox7a2, Cox7c, Cox8a, Ndufa2, Ndufa4, Uqcrh, Uqcrq] |
| GO:0019646 | aerobic electron transport chain | 6.52 | Group08 | 3 | [Cox7c, Uqcrh, Uqcrq] |
| GO:0042775 | mitochondrial ATP synthesis coupled electron transport | 5.08 | Group08 | 3 | [Cox7c, Uqcrh, Uqcrq] |
| GO:0015078 | proton transmembrane transporter activity | 6.52 | Group08 | 9 | [Atp5e, Atp5g1, Atp5g2, Atp5h, Atp5l, Cox7a2, Cox7c, Cox8a, Ndufa4] |
| GO:0004129 | cytochrome-c oxidase activity | 12.12 | Group08 | 4 | [Cox7a2, Cox7c, Cox8a, Ndufa4] |
| GO:0009205 | purine ribonucleoside triphosphate metabolic process | 6.98 | Group08 | 6 | [Atp5e, Atp5g1, Atp5g2, Atp5h, Atp5l, Ran] |
| GO:0015662 | P-type ion transporter activity | 6.52 | Group08 | 3 | [Atp5e, Atp5l, Vmp1] |
| GO:0015986 | ATP synthesis coupled proton transport | 22.73 | Group08 | 5 | [Atp5e, Atp5g1, Atp5g2, Atp5h, Atp5l] |
| GO:0046933 | proton-transporting ATP synthase activity, rotational mechanism | 18.75 | Group08 | 3 | [Atp5e, Atp5h, Atp5l] |
| GO:1990948 | ubiquitin ligase inhibitor activity | 58.33 | Group09 | 7 | [Rpl11, Rpl23, Rpl37, Rpl5, Rps15, Rps20, Rps7] |
| GO:0072331 | signal transduction by p53 class mediator | 8.55 | Group09 | 13 | [Mif, Npm1, Rpl11, Rpl23, Rpl26, Rpl37, Rpl5, Rps15, Rps20, Rps27a, Rps27l, Rps7, Ubc] |
| GO:0008630 | intrinsic apoptotic signaling pathway in response to DNA damage | 4.2 | Group09 | 5 | [Mif, Rpl26, Rps27l, Rps3, Tpt1] |
| GO:0030330 | DNA damage response, signal transduction by p53 class mediator | 5.8 | Group09 | 4 | [Mif, Npm1, Rpl26, Rps27l] |
| GO:0072332 | intrinsic apoptotic signaling pathway by p53 class mediator | 8.43 | Group09 | 7 | [Mif, Rpl11, Rpl26, Rps27a, Rps27l, Rps7, Ubc] |
| GO:1901796 | regulation of signal transduction by p53 class mediator | 14.63 | Group09 | 12 | [Mif, Npm1, Rpl11, Rpl23, Rpl26, Rpl37, Rpl5, Rps15, Rps20, Rps27a, Rps7, Ubc] |
| GO:0043516 | regulation of DNA damage response, signal transduction by p53 class mediator | 8.11 | Group09 | 3 | [Mif, Npm1, Rpl26] |
| GO:1901798 | positive regulation of signal transduction by p53 class mediator | 25.71 | Group09 | 9 | [Rpl11, Rpl23, Rpl26, Rpl37, Rps15, Rps20, Rps27a, Rps7, Ubc] |
| GO:2001242 | regulation of intrinsic apoptotic signaling pathway | 5.59 | Group09 | 10 | [Mif, Ppia, Rack1, Rpl11, Rpl26, Rps27a, Rps3, Rps7, Tpt1, Ubc] |
| GO:0042771 | intrinsic apoptotic signaling pathway in response to DNA damage by p53 class mediator | 6.52 | Group09 | 3 | [Mif, Rpl26, Rps27l] |
| GO:0045727 | positive regulation of translation | 5.59 | Group09 | 8 | [Eif5a, Npm1, Rpl26, Rpl5, Rps27l, Rps3a1, Rps4x, Rps9] |
| GO:1902229 | regulation of intrinsic apoptotic signaling pathway in response to DNA damage | 10.26 | Group09 | 4 | [Mif, Rpl26, Rps3, Tpt1] |
| GO:2001244 | positive regulation of intrinsic apoptotic signaling pathway | 9.72 | Group09 | 7 | [Rack1, Rpl11, Rpl26, Rps27a, Rps3, Rps7, Ubc] |
| GO:1902253 | regulation of intrinsic apoptotic signaling pathway by p53 class mediator | 17.65 | Group09 | 6 | [Mif, Rpl11, Rpl26, Rps27a, Rps7, Ubc] |
| GO:0021884 | forebrain neuron development | 7.32 | Group09 | 3 | [Rps27a, Ubc, Uqcrq] |
| GO:0031396 | regulation of protein ubiquitination | 5.8 | Group09 | 13 | [Npm1, Ppia, Rpl11, Rpl23, Rpl37, Rpl5, Rps15, Rps2, Rps20, Rps27a, Rps3, Rps7, Ubc] |
| GO:0051438 | regulation of ubiquitin-protein transferase activity | 13.11 | Group09 | 8 | [Rpl11, Rpl23, Rpl37, Rpl5, Rps15, Rps2, Rps20, Rps7] |
| GO:0031397 | negative regulation of protein ubiquitination | 10.11 | Group09 | 9 | [Ppia, Rpl11, Rpl23, Rpl37, Rpl5, Rps15, Rps20, Rps3, Rps7] |
| GO:2000059 | negative regulation of ubiquitin-dependent protein catabolic process | 7.14 | Group09 | 4 | [Rpl11, Rpl23, Rpl5, Rps7] |
