# Supplementary figures and images for: Single-cell transcriptomic analysis reveals a novel cell state and switching genes during hepatic stellate cell activation in vitro
Source: J Transl Med. 2022 Jan 29;20:53. doi: 10.1186/s12967-022-03263-4 (PMC8800312; doi:10.1186/s12967-022-03263-4)

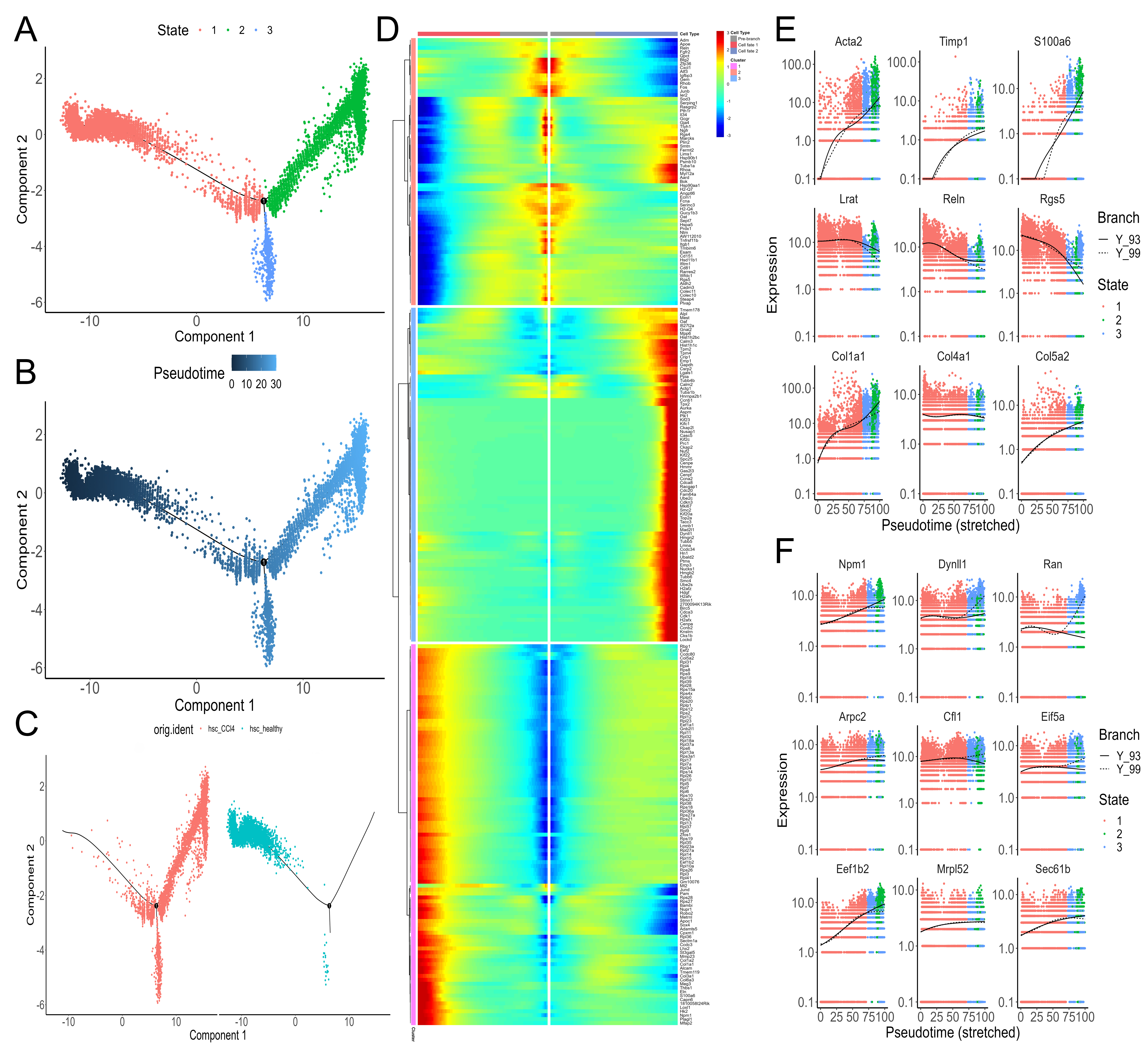

Supplement: Supplementary file 4 — Additional file 4: Fig. S1. Simulation of the differentiation trajectory of HSCs isolated from healthy and CCl4-treated mouse liver and the analysis of gene expression pattern. [file 12967_2022_3263_MOESM4_ESM.jpg]

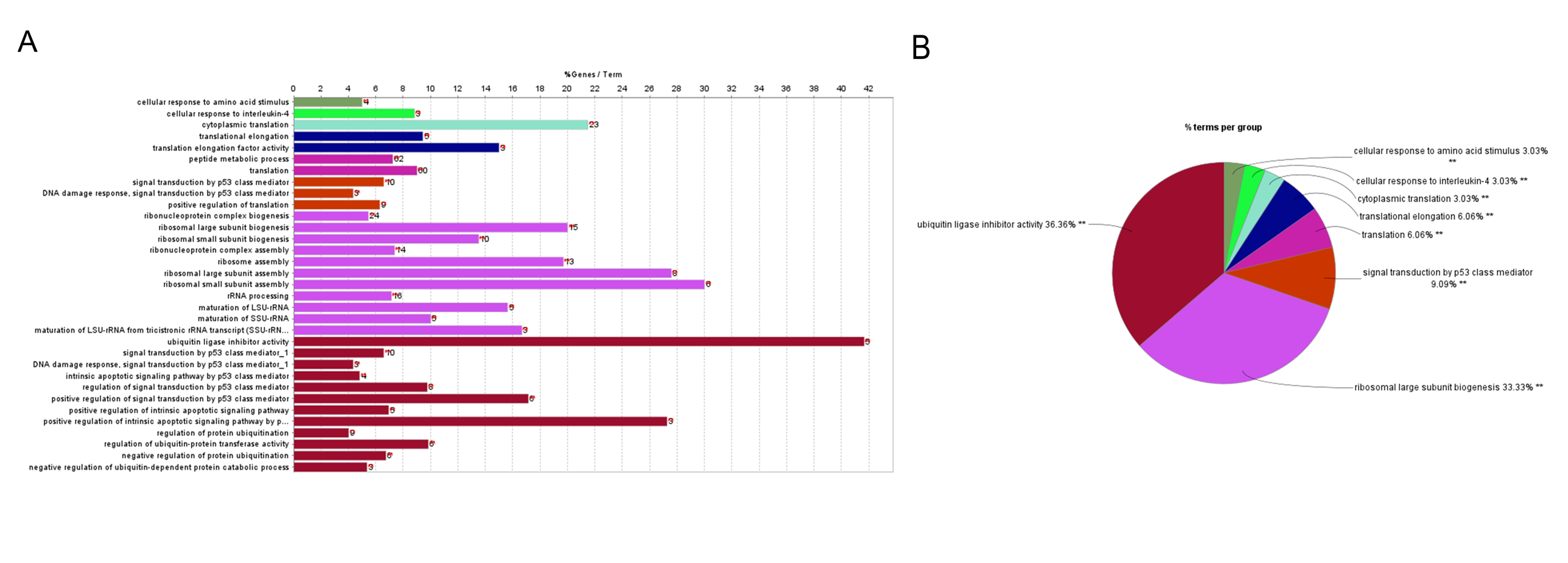

Supplement: Supplementary file 5 — Additional file 5: Fig. S2. ClueGO and CluePedia were used for biological process annotation of the genes in cluster 3 of the branched heatmap (Fig. S1D) for HSC differentiation trajectory. [file 12967_2022_3263_MOESM5_ESM.jpg]

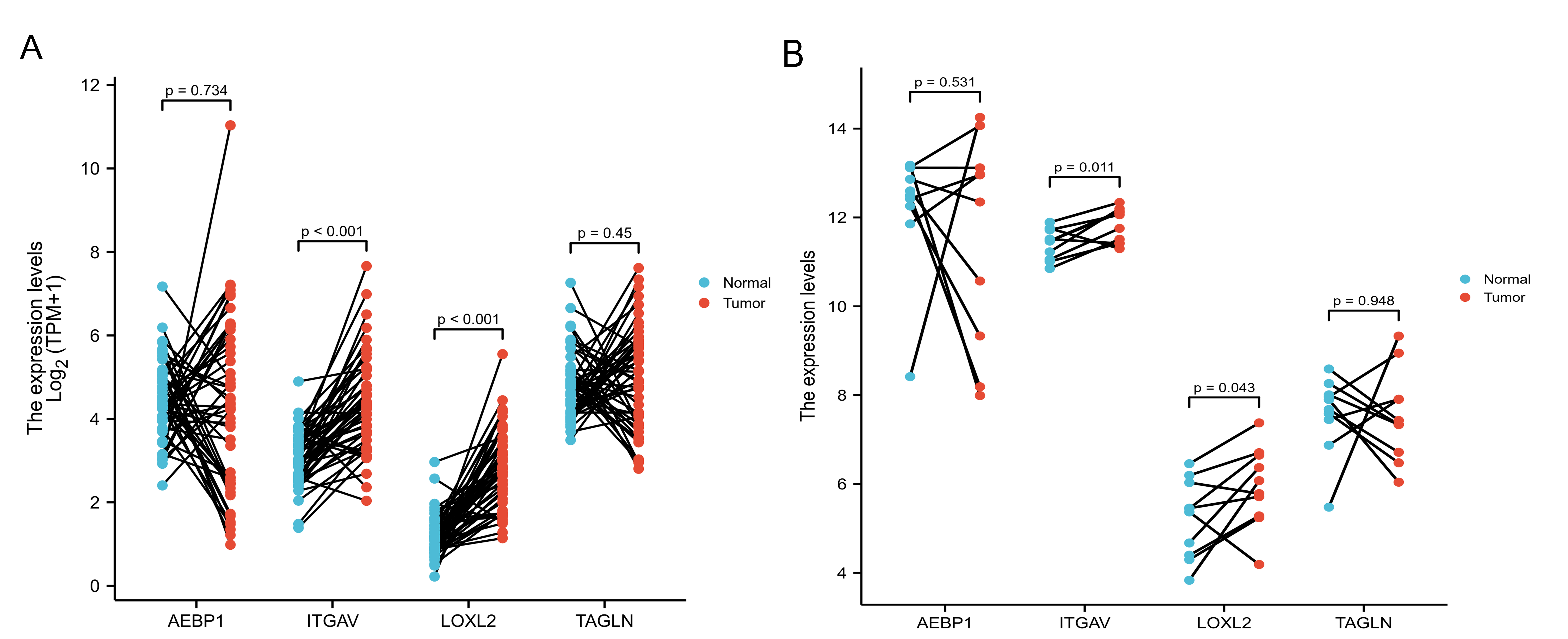

Supplement: Supplementary file 6 — Additional file 6: Fig. S3. Comparison of the predictive fibrosis markers expressed in HCC and normal tissues. [file 12967_2022_3263_MOESM6_ESM.jpg]

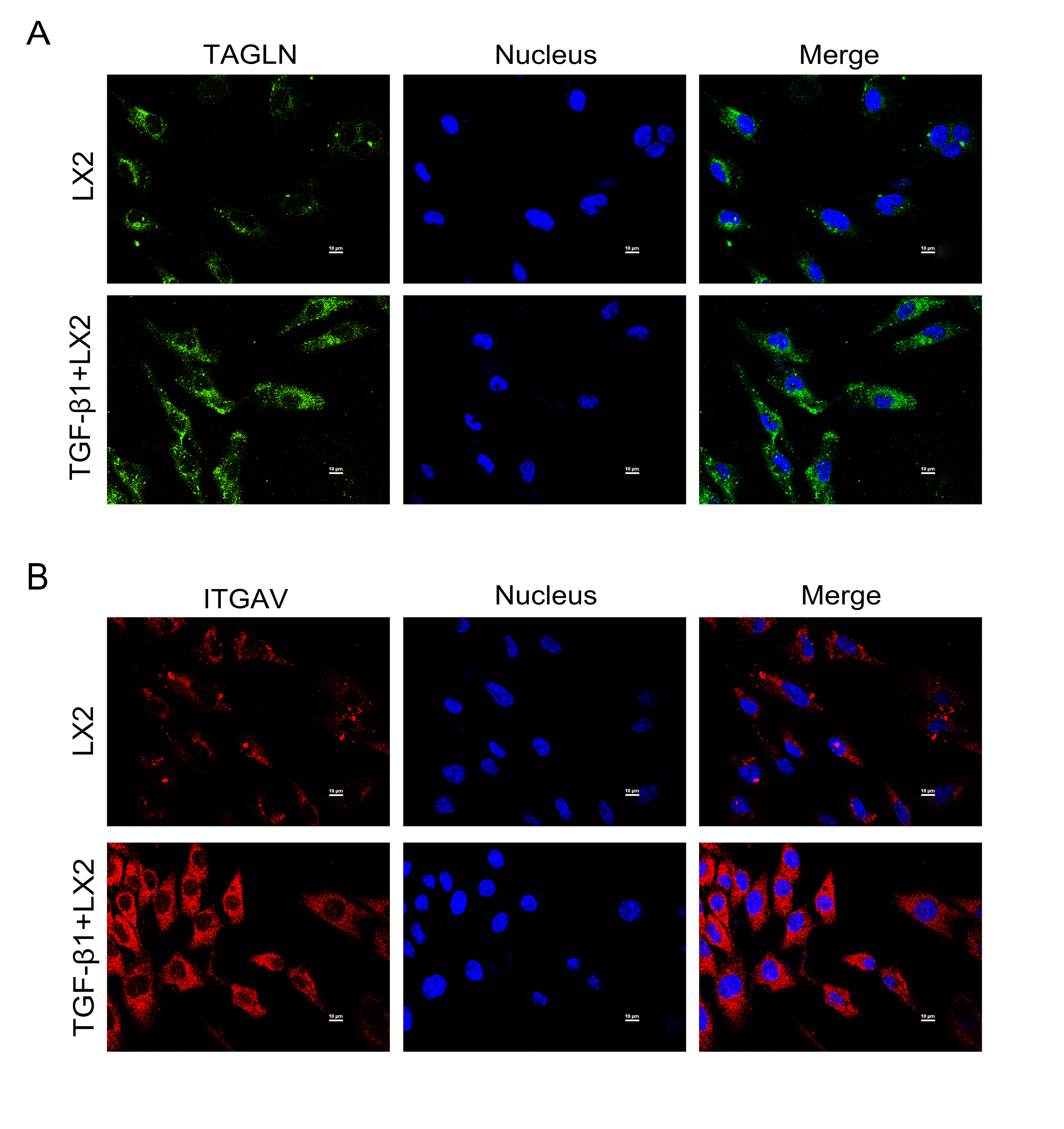

Supplement: Supplementary file 8 — Additional file 8: Fig. S5. Experimental verification of the expression of TAGLN and ITGAV in human hepatic stellate LX2 cells. [file 12967_2022_3263_MOESM8_ESM.jpg]
